# A genome-wide CRISPR/Cas9 screen identifies DNA-PK as a sensitiser to ^177^Lutetium-DOTA-octreotate radionuclide therapy

**DOI:** 10.7150/thno.84628

**Published:** 2023-08-28

**Authors:** Kelly Waldeck, Jessica Van Zuylekom, Carleen Cullinane, Twishi Gulati, Kaylene J. Simpson, Richard W. Tothill, Benjamin Blyth, Rodney J. Hicks

**Affiliations:** 1 Models of Cancer Translational Research Centre, Research Division, Peter MacCallum Cancer Centre, 305 Grattan St, Melbourne, Victoria, Australia, 3000.; 2 Sir Peter MacCallum Department of Oncology, The University of Melbourne, Parkville, Victoria, Australia, 3010.; 3 Victorian Centre for Functional Genomics, Peter MacCallum Cancer Centre, 305 Grattan St, Melbourne, Victoria, Australia, 3000.; 4 Department of Biochemistry and Pharmacology, The University of Melbourne, Parkville, Victoria, Australia, 3010.; 5 Department of Clinical Pathology and University of Melbourne Centre for Cancer Research, The University of Melbourne, Parkville, Victoria, Australia, 3010.; 6 St Vincent's Hospital Department of Medicine, The University of Melbourne, Parkville, Victoria, Australia, 3010.

**Keywords:** PRRT, DNA-PK, CRISPR, somatostatin receptor, radionuclide therapy

## Abstract

Peptide receptor radionuclide therapy (PRRT) using ^177^Lutetium-DOTA-octreotate (LuTate) for neuroendocrine tumours (NET) is now an approved treatment available in many countries, though primary or secondary resistance continue to limit its effectiveness or durability. We hypothesised that a genome-wide CRISPR/Cas9 screen would identify key mediators of response to LuTate and gene targets that might offer opportunities for novel combination therapies for NET patients.

**Methods:** We utilised a genome-wide CRISPR-Cas9 screen in LuTate-treated cells to identify genes that impact on the sensitivity or resistance of cells to LuTate. Hits were validated through single-gene knockout. LuTate-resistant cells were assessed to confirm LuTate uptake and retention, and persistence of somatostatin receptor 2 (SSTR2) expression. Gene knockouts conferring LuTate sensitivity were further characterised by pharmacological sensitisation using specific inhibitors and *in vivo* analysis of the efficacy of these inhibitors in combination with LuTate.

**Results:** The CRISPR-Cas9 screen identified several potential targets for both resistance and sensitivity to PRRT. Two gene knockouts which conferred LuTate resistance *in vitro*, *ARRB2* and *MVP*, have potential mechanisms related to LuTate binding and retention, and modulation of DNA-damage repair (DDR) pathways, respectively. The screen showed that sensitivity to LuTate treatment *in vitro* can be conferred by the loss of a variety of genes involved in DDR pathways, with loss of genes involved in Non-Homologous End-Joining (NHEJ) being the most lethal. Loss of the key NHEJ gene, *PRKDC* (DNA-PK), either by gene loss or inhibition by two different inhibitors, resulted in significantly reduced cell survival upon exposure of cells to LuTate. In SSTR2-positive xenograft-bearing mice, the combination of nedisertib (a DNA-PK specific inhibitor) and LuTate produced a more robust control of tumour growth and increased survival compared to LuTate alone.

**Conclusions:** DDR pathways are critical for sensing and repairing radiation-induced DNA damage, and our study shows that regulation of DDR pathways may be involved in both resistance and sensitivity to PRRT. Additionally, the use of a DNA-PK inhibitor in combination with LuTate PRRT significantly improves the efficacy of the treatment in pre-clinical models, providing further evidence for the clinical efficacy of this combination.

## Introduction

Peptide receptor radionuclide therapy (PRRT) is now available in many countries for the treatment of neuroendocrine tumours (NET). Regulatory approval was largely based on the positive outcome of the NETTER-1 randomised control trial in patients with low/intermediate grade small intestinal NET 1. However, broader application reflects a large body of experience from institutions around the world. Beneficial outcomes and low toxicity of PRRT have now been described in NET arising from other primary sites 2, of higher grade 3 and in other diseases that express the therapeutic target, the somatostatin 2 receptor (SSTR2), including phaeochromocytoma/paraganglioma 4-6 and neuroblastoma 7.

Despite the proven effectiveness of PRRT, and the ability to select patients based on high uptake of ^68^Galium-DOTA-octreotate (GaTate) on PET imaging, which is suggestive of high expression of SSTR2, objective response rates of only 20-30% have been reported with around 10% of patients demonstrating progressive disease during treatment 2, 8. Even in patients who initially respond well, later progression is often observed, and although retreatment can be effective 9, many of these patients develop refractory disease. Clearly there is ample scope to improve on the efficacy of PRRT. Approaches to achieve this goal have included increasing the administered activity, in the expectation of achieving higher radiation dose delivery 10, and concurrent use of radiosensitising chemotherapy 11-13. However, the degree to which these approaches have been effective remains unclear and new strategies are needed to deal with PRRT refractory disease.

The primary mechanism of disease control from radionuclide therapy is assumed to be DNA-damage. Having been exposed to terrestrial and cosmic radiation for eons, mammalian cells have evolved complex and somewhat overlapping mechanisms of DNA-damage repair (DDR) 14. While these mechanisms serve to maintain the integrity of the genetic code after radiation exposure, they also act to limit the efficacy of therapeutic irradiation. Accordingly, DDR-modifying drugs may be an attractive approach to alter the therapeutic efficacy of PRRT. Beta-particle radiation, like that resulting from the most widely used agent for PRRT, ^177^Lutetium-DOTA-octreotate (LuTate, Lutathera®, NOVARTIS) is thought to result in the generation of substantially more single-strand DNA breaks (SSB) than double-strand DNA breaks (DSB). The pathways within cells that repair these two types of DNA damage are different, but complementary. Base Excision Repair (BER) is the predominant pathway for the repair of SSB, while DSB repair can proceed through multiple pathways, including Homologous Recombination (HR) and Non-Homologous End Joining (NHEJ) 15, 16. Research around combining DDR inhibitors with PRRT has focussed significantly on PARP-inhibitors, which act to block repair of SSB. *In vitro* cell studies suggest that PARP inhibition increases cell killing in combination with LuTate 17, supporting prior results involving *ex vivo* culture of NET slices treated with PRRT in combination with the PARP-inhibitor, olaparib 18. Our group extended this approach, demonstrating enhanced response and survival of xenograft-bearing mice with the combination of PRRT and the PARP inhibitor talazoparib 19, which has now entered into a phase I clinical trial (PARLuNET, NCT05053854). While this approach targets SSB, other DDR-modifying drugs that are entering clinical trials, especially agents that impact on repair of DSB 15, may also prove efficacious.

While regulation of DDR pathways is important in regulating the effectiveness of PRRT, we recognised that there are likely many other genetic factors also influencing response to LuTate PRRT. As such, we performed a genome-wide CRISPR-Cas9 knockout screen to identify novel gene targets to elucidate differential responses to PRRT and inform the development of novel combination therapies for NET patients.

## Methods

### Cell lines, Drugs and PRRT

H1299-7 cells, constitutively overexpressing SSTR2 (a gift from Buck Rogers, Mallinckrodt Institute, St Louis, Missouri), AR42J cells expressing high SSTR2 and SKNBE-2 expressing low levels of SSTR2, are as described in Cullinane *et al* 19. The identity of the H1299-7 and SKNBE-2 cell lines was confirmed through STR profiling (AGRF, Melbourne, Australia). Nedisertib and AZD-7648 were purchased from Med Chem Express. LuTate was produced using [DOTA^0^, Tyr^3^] octreotate and carrier-added [^177^Lu] Lutetium chloride (IDB, the Netherlands).

### Generation of Cas9 expressing cells

H1299-7 cells were transduced with FUCas9Cherry vector (plasmid #70182, Addgene), using standard protocols. The top 20-30% of mCherry expressing cells were sorted by FACS resulting in the H1299-7 Cas9 cell line. To assess Cas9 editing efficiency, H1299-7 Cas9 cells were transduced with the GFP containing pXPR-011 construct (Plasmid #59702, Addgene 20), and mCherry and GFP expression assessed by flow cytometry at 7 days post-transduction. Cas9 editing efficiency was determined by the loss of GFP expression in the Cas9 cells, as compared to control cells, with loss of GFP equating to greater editing efficiency.

### Genome-wide CRISPR-Cas9 screen

H1299-7 Cas9 cells were transduced with the Human Brunello CRISPR knockout pooled library (#73178, Addgene 21), obtained through the Victorian Centre for Functional Genomics (VCFG) at the Peter MacCallum Cancer Centre. H1299-7 Cas9 cells were expanded and the day prior to transduction twenty 175 cm^2^ flasks were plated at 10 × 10^6^ cells per flask. On the day of transduction, one 175 cm^2^ flask was counted, and virus volume required per flask calculated:



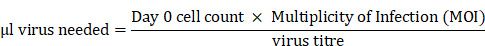



with virus titre having been previously determined using standard protocols.

Next, 200-250 × 10^6^ cells were transduced with the Brunello library at an MOI of 0.3. Cells were transduced in the presence of 8 μg/mL sequebrene (Sigma-Aldrich) in 20 mL of media for 16 h. Cells were then trypsinised, pooled and replated, and treated with 2.5 μg/mL puromycin (Sigma-Aldrich) for 96 h to select for successfully transduced cells.

A baseline sample of transduced cells (40 × 10^6^ cells, representing 500× coverage for the Brunello library) was collected prior to treatment. For LuTate treatment, 160 × 10^6^ cells (at 8 × 10^6^ cells/mL in DMEM + 1%FCS) were incubated in suspension with 5 MBq/mL LuTate for 4 h at 37°C and 5% CO_2_, with resuspension/mixing every hour. After 4 h, cells were washed and resuspended in 16 mL of full media [DMEM + 10% FCS + 1% Non-Essential Amino Acids (Gibco) + 1% Sodium Pyruvate (Gibco)] and plated in 175 cm^2^ flasks at 10 × 10^6^ cells. For the untreated controls, 80 × 10^6^ cells were handled in the same manner before plating out. Since a CRISPR-Cas9 screen such as this has not previously been described with PRRT, the best parameters for the collection of samples were unknown. As such, for the first replicate of the screen, samples were collected across timepoints representing early in treatment and three subsequent half-lives of the LuTate PRRT (where the half-life of Lutetium^177^ is 6.7 days). At each of the chosen timepoints (Days 2, 7, 14, and 21) 40 × 10^6^ cells were collected from both treated and untreated arms of the screen, with the remaining cells replated. Analysis of the initial screen showed that the most significant results were gained at Day 21, and as such the screen was repeated with only baseline and Day 21 samples collected.

DNA was extracted from frozen cell pellets using the QIAGEN Blood and Cell Culture DNA Maxi kit, as per manufacturer's instructions (#13362, Qiagen). Sample concentration was determined by nanodrop, and PCR reactions carried out with long primers incorporating adapters necessary for Illumina sequencing (P5 and P7 primers obtained from the VCFG, primer details in supplementary Table 1), using standard protocols. For each sample a total of 80 μg of DNA was PCR amplified. PCR reactions set up as below:

PCR run parameters were: 95°C for 1 min; 26 cycles of 95°C for 30 sec, 53°C for 30 sec, 72°C for 30 sec; and finally, 10 min at 72°C. Individual PCR reactions were then pooled per sample, and purified using AMPure XP magnetic beads (Beckman Coulter, Australia), as per standard protocols. Samples were sequenced using Illumina NextSeq500 with 20-40 million reads/sample (Molecular Genomics Core, Peter MacCallum Cancer Centre).

### CRISPR-Cas9 screen analysis and Hit Identification

FASTQ data files from the screen were analysed using a pipeline established in Galaxy (usegalaxy.org.au). Files were trimmed to remove adapter sequences using Cutadapt 1.16 with Python 3.6.5. Single-end reads were trimmed and resulting files processed using Model-based Analysis of Genome-wide CRISPR/Cas9 Knockout (MAGeCK) tools 22. MAGeCK count (Galaxy version 0.5.9.2.4) was used to collect sgRNA read counts, aligning gRNA sequences from the Brunello library (accessed from Addgene.org) with the sequenced data. MAGeCK test (Galaxy version 0.5.9.2.1) analyses were carried out on the sgRNA count files generated. Each test sample (treated and untreated at Days 2, 7, 14 and 21) was tested against the baseline sample, giving an output of gene and sgRNA summary ranking, and p-values for both resistance (enrichment, positive selection screen) and sensitivity (depletion, negative selection screens). The p-values across both replicates of the screen were averaged, reducing the likelihood of false positives being identified within the screen. Results were visualised with GraphPad Prism. Pathway analysis was carried out using a PANTHER overrepresentation test (pantherdb.org), with the human genome as the reference set, GO biological processes as the annotation, undertaking Fisher's exact test, and calculation of false discovery rate as the correction. Gene 'hits' for resistance and sensitivity were identified as genes that had a significant p-value in the Day 21 LuTate-treated samples as compared to baseline (p-value of < 0.005) and were not significant in the Day 21 untreated samples as compared to baseline (p-value > 0.05).

### Single Gene knockout

Two gRNA (from the Brunello library, as determined in the MaGeCK count files) with the most significant enrichment or depletion for the genes to be validated, were pooled and nucleofected into H1299-7 Cas9 cells, using the Amaxa 4D nucleofector (Lonza), solution SF and program EW-127 (Lonza, optimised protocol for H1299 cells). Cells were nucleofected with 75 pmol of each gRNA in 20 μl (gRNA from Integrated DNA Technologies, sequences in supplementary Table 2). Efficiency of gene knockout was determined at 72 h post nucleofection through western blot or DNA sequencing. The resulting cell lines were H1299-7 Beta-Arrestin 2 knockout (KO), MVP KO, Artemis KO, and DNA-PK KO.

### Western Blot

Cell Pellets (1-2 × 10^6^) were lysed in 150 μl RIPA buffer containing phosphatase and protease inhibitors using standard protocols, with protein concentration determined using Pierce BCA Protein Assay kit (Thermo-Fischer Scientific) as per manufacturer's instructions. Samples were separated on 10% SDS-Page gels, and for DNA-PK blots 5% - 12% gradient SDS-page gels (Mini-protean TGX gels, BioRad). Gel electrophoresis and transfer were carried out using standard protocols. PVDF membranes (Thermo-Fischer Scientific) were blocked and probed overnight with primary antibody; MVP (1:1000 ab273093, Abcam), Beta-Arrestin 2 (1:1000 ab54790, Abcam), Ku-70 (1:1000 #4588, Cell Signalling), Ku-80 (1:1000 #2180, Cell Signalling), Bcl-2 (1:1000 #2870, Cell Signalling), DNA-PK (1;1000 MA5-32192, Invitrogen) or Actin (1:10000 #A2228 Sigma). Membranes were incubated with the appropriate secondary antibodies for 1 h at room temperature (RT), before 2 min incubation with Clarity Western ECL substrate (#170-5060, BioRad), and visualisation on the ChemiDoc Imaging System (BioRad).

### DNA Sequencing

Cell Pellets (1-2 × 10^6^) were collected, and DNA extracted using the Qiagen DNA extraction kit (QIAamp DNA Mini Kit #56304, Qiagen), as per manufacturer's instructions. The region flanking the CRISPR cut site was amplified by PCR using gene specific primers (Supplementary Table 3), and sequenced by Sanger Sequencing (AGRF, Melbourne Australia). Analysis was performed through sequence alignment looking for insertions/deletions within the CRISPR guide region.

### Cell Growth Assays

For LuTate treatment, aliquots of 4 × 10^6^ cells were collected and resuspended in 500 μl DMEM + 1% FCS, and treated with either 0, 5, 10 or 20 MBq/mL LuTate for 4 h in suspension at 37°C and 5% CO_2_ with mixing every hour. Cells were resuspended in 4 mL full media and plated in 6 well plates at a concentration of 0.5 × 10^6^ cells per well. For drug combination experiments, 24 h post LuTate treatment, cells were treated with either 0.5 μM or 1 μM of either AZD-7648 or nedisertib.

Cell growth was tracked over a 14-day period. Cells were counted and replated at 0.2 × 10^6^ cells per well on Days 2, 6 and 9, with a final count on Day 14. Because of the exponential growth or significant lethality of the various cultures, not all cells were necessarily re-seeded at each subculture interval. However, the cell growth assay data is corrected for the fraction of cells re-plated so the data is expressed for all conditions as though all cells were re-plated at each interval (illustrated in Supplementary Figure S1). The total number of cells is thus the theoretical total number of cells over time if all cells at each subculture interval were re-seeded at identical density, calculated by:

Total Cells = Cell Number counted (1 well) × Number of Wells required

Number of Wells required going forward = Total Cells/0.2 × 10^6^

Cell growth was normalised to the appropriate control for each experiment. For growth curves showing validation of the CRISPR result, results were graphed showing the alteration in cell population, as compared to control, over time (out to Day 14). For all other assays (combination experiments, and experiments using AR42J or SKNBE-2 cells), results are shown comparing cell population at the Day 14 timepoint. All assays were repeated as at least biological triplicates.

### LuTate Retention Assays

H1299-7, H1299-7 Cas9, MVP KO and Beta-Arrestin 2 KO cells were treated with either 0 or 5 MBq/mL LuTate using the same suspension protocol as for the growth experiments. After 4 h of treatment, cells were washed and triplicate aliquots collected for the 4 h timepoint, and the remaining cells plated in triplicate in 6 well plates for 24 h. For collection of samples, cells were spun down, washed twice, and resuspended in 500 μl PBS. All experiments were repeated as biological triplicates. Samples were stored at RT for 2-3 half-lives of LuTate (2-3 weeks), before being counted on the Captus 4000e gamma counter (Capintec). Percent LuTate retained by the cells was calculated as a percent of total activity added and averaged across the triplicate experiments.

### Immunofluorescence staining

H1299-7, H1299-7 Cas9, MVP KO and Beta-Arrestin 2 KO cells were treated with 0 or 5 MBq/mL LuTate using the same suspension protocol as for the growth assays. After treatment cells were plated in 6 well plates for 24 h before being collected, washed, and fixed in 4% paraformaldyde for 20 min at 4°C. Cells were stored in 70% ethanol at 4°C for 2-3 weeks (2-3 half-lives of LuTate).

For staining, cells were brought to RT, resuspended in PBS, and spun at 400 rpm for 8 min onto glass slides. Slides were blocked (PBS + 8% BSA) for 30 min, before staining with primary SSTR2 antibody for 1 h at RT (1:200 dilution, Ab134152, Abcam), followed by the anti-rabbit HRP (1:1000, Bio-Rad) secondary antibody for 1 h at RT. Slides were then further incubated for 10 min with TSA 520 reagent (Akoya Biosciences), counterstained with Dapi, and imaged on a BX-53 microscope, with a Nikon camera at 40× and 20× magnification.

### *In vivo* Experiments

All animal experiments were performed with approval from the Peter MacCallum Cancer Centre Animal Experimentation Ethics Committee, and in accordance with the Australian code for the care and use of animals for scientific purposes (8th Edition, 2013). Four- to seven-week-old female BALB/c nude mice were sourced from Animal Resources Centre (Canning Vale, Western Australia). 3 × 10^6^ AR42J or 5 × 10^6^ H1299-7 cells were subcutaneously implanted onto the right flank of the mice in a 50% Matrigel (Corning):PBS mixture. Mice were weighed, and tumours measured twice weekly using electronic calipers. Tumour volume (mm^3^) was calculated as length × width × height × π/6. Once tumours reached a volume of 50-300 mm^3^, the animals were randomised into groups of 9-10 mice and injected intravenously with a single dose (5-10 MBq in 100-200 μL) LuTate or vehicle (saline). At 24 h post LuTate injection mice began once-daily oral dosing of DNA-PK inhibitor nedisertib (150 mg/kg) or vehicle (10% DMSO, 40% PEG300, 5% Tween-80, 45% saline) in a volume of 10 ml/kg for 6 consecutive days. Mice were monitored 2-3 times weekly for overall weight as an indicator of murine health and tumour volume measured. Mice were euthanised once the tumour volume exceeded 1200 mm^3^.

## Results

**Figure 1:**
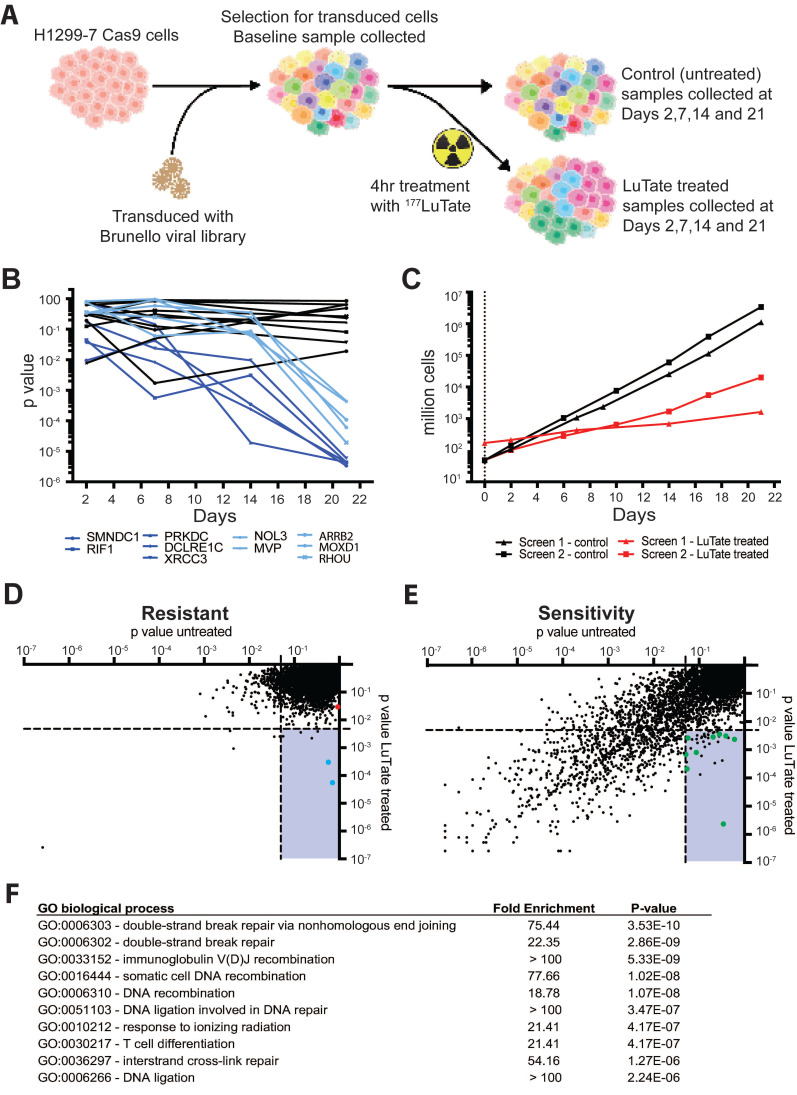
A genome-wide CRISPR-Cas9 knockout screen identifying genes that impact resistance or sensitivity to LuTate. **A)** Schematic of the screen set up using H1299-7 cells and the Brunello CRISPR sgRNA library. **B)** Change in p-value over time for the top 10 gene hits, five each from the resistance and sensitivity arms of the initial replicate of the screen. Control p-values for the 10 genes are in black, with the LuTate treated values in dark blue (for the sensitivity arm of the screen) and light blue (for the resistance arm of the screen). **C)** Comparison of growth rates for control and LuTate treated cells over the course of 21 days of the screen, in the two replicates of the screen. **D and E)** Plots of individual genes of the resistance arm (D) and sensitivity arm (E) of the screen, showing averaged p-value data from the two replicates at Day 21. Data is visualised as the comparison between control untreated p-values (on the X axis) and LuTate treated p-values (on the Y axis). The lower right quadrant in both plots contains genes significantly altered in response to LuTate (p-value <0.005), but unaltered in the control cells (p-value >0.05). In the resistance arm of the screen (D) blue dots are the two most significant hits, *MVP* and *ARRB2* (Beta-Arrestin 2), the red dot is *SSTR2*. In the sensitivity arm of the screen (E) green dots are those genes that, as members of the Non-Homologous End-Joining (NHEJ) DNA-strand break repair pathway, contributed to this pathway being the most significant GO biological process pathway identified **(F)**.

To identify novel modulators of response to PPRT, we undertook a genome-wide screen to identify potential targets without the need for *a priori* selection of candidate genes or pathways 21, 23. The H1299-7 human cell line was chosen for the screen as it is known to stably express high levels of SSTR2 (reducing interference from heterogeneous target expression) and is responsive to LuTate treatment in culture and as a tumour xenograft 19. Other cell lines commonly used to study LuTate PRRT are either of non-human origin (i.e. the rat AR42J cells for which there is no validated genome-wide screen available) or respond poorly to LuTate *in vitro* making them unsuitable for a large screening assay. The screen assessed CRISPR-Cas9 induced loss of gene function that resulted in either increased LuTate resistance (enrichment of cells harbouring a gRNA) or increased LuTate sensitivity (depletion of cells harbouring a gRNA) when a very large pool of cells carrying a diversity of single gene disruptions across the genome were treated with LuTate (Figure 1A).

The design of our CRISPR screen had to consider several factors that are unique to radionuclide therapy, and required significant optimisation. Optimisation of the conditions for the screen are outlined in supplementary Figure S2, including determination of Cas9 editing efficiency (Figure S2A), and the optimal cell numbers and LuTate activity required for a 50-70% reduction in cell survival after PRRT treatment (Figure S2B and S2C). CRISPR-Cas9 screens are commonly assayed at timepoints out to 21 days, allowing time for phenotypic changes as the result of genetic editing to become apparent 24, 25. In practice, two genome-wide screens were performed side-by-side, one with no treatment and the other with a single LuTate treatment, both then expanded for 21 days. In the first replicate of our side-by-side screens, the change in gRNA representation in the cell population was sampled over four consecutive timepoints - Days 2, 7, 14 and 21 - in both LuTate-treated and untreated screens, and the resulting representation of gRNA compared to their respective baselines. Time course data of gRNA representation (by p-value, taking into account the degree of enrichment/depletion across 4 gRNA per gene) for the ten most significantly altered genes - five genes each from the resistance and sensitivity arms of the screen - showed that enrichment or depletion of the gRNA's was most significant at Day 21, and that the degree of enrichment/depletion increased over time throughout the screen (Figure 1B). This is consistent with the idea of early mechanisms (e.g. short-term radiation-induced cell death) maintaining early depletion/enrichment throughout the assay, and longer-term mechanisms (e.g. persistently altered growth rates) showing increasing depletion/enrichment as the whole population continues to expand. Therefore, the second independent replicate of the side-by-side screens (which showed similar growth-rates and LuTate response as the first, Figure 1C) was sampled at only baseline and Day 21.

The screen data is therefore presented as the p-value of the untreated Day 21 screen versus the LuTate-treated Day 21 screen, averaged over the two independent replicate screens, and plotted separately for both the resistance (Figure 1D) and the sensitivity (Figure 1E) arms of the screen. Each point on the plot represents an individual gene, and each gene is present in both plots as the evidence for enrichment and the evidence for depletion are calculated for each gene producing a statistical score for both mechanisms (full data in supplementary Table 4). As expected, in both the LuTate-treated and untreated cells at Day 21, most genes showed no significant enrichment or depletion of the gRNA's and therefore had no significant impact on cell growth (upper right quadrants in Figure 1D and 1E, respectively). The lower right quadrant of each plot shows single-gene knockouts that had a significant effect on cell growth/survival (and hence gRNA representation) in response to LuTate treatment (using a strict p-value cut-off of <0.005 for inclusion), while showing no effect in the untreated samples (using a more relaxed p-value threshold of >0.05 for exclusion). This provided our candidate LuTate specific gene list. Knockout of single genes was more likely to reduce cell growth, with or without LuTate treatment, than to provide a growth advantage. This is also seen in the LuTate specific gene candidates - where more genes were identified as resulting in sensitivity than in resistance (39 genes, as compared to 6, lower right quadrant in Figure 1D and 1E).

As an internal control, since loss of *SSTR2* would theoretically be the most direct way to result in resistance to LuTate, we assessed the impact of the loss of *SSTR2* within the screen data. *SSTR2* loss significantly conferred resistance (Figure 1E, red dot in plot), even though it didn't meet the strict p-value thresholds we set to be included as a resistance candidate (p-value 0.029 > 0.005). This suggests that our genome-wide screen was operating as designed, particularly given that in the specific case of *SSTR2* the H1299-7 cell line carries multiple additional copies of the *SSTR2* gene 26 such that the CRISPR-Cas9 knockout may not result in complete gene-editing in each cell. In addition, mechanisms that simply prevent LuTate binding are biased against in the screen, since cross-fire from LuTate bound to neighbouring cells will ensure all cells are irradiated to a substantial degree even if LuTate binding to a given cell is significantly reduced. While there were no biological pathways enriched by Gene Ontology (GO) pathway analysis in the 6 selected candidates from the resistance arm of the screen, two highly significant genes were identified, *ARRB2* (coding for Beta-Arrestin 2) and *MVP* (Major Vault Protein) (blue dots in 1E).

Of the 39 candidate genes selected as being LuTate-specific in the sensitivity arm of the screen, most had a clear functional relationship to radiation-induced lethality. GO pathway analysis identified the Double-Strand DNA Break (DSB) Repair via Non-Homologous End-Joining (NHEJ) as the most significant pathway (Figure 1F). Of the 39 candidate sensitivity genes, 9 were within the NHEJ pathway, including *PRKDC*, *DCLRE1C*, *XRCC4*, *LIG4* and *NHEJ1* (green dots in Figure 1D); with the single most significant gene for sensitivity to LuTate being *PRKDC* (coding for DNA-PK, p-value 0.00000233). Other DNA repair candidates were also identified, most of which were highly specific to key nodes within a given repair pathway, such as *RAD51B* in Homologous Recombination (HR). The sensitivity arm of the screen also identified some genes that aren't known to be involved in DDR pathways, genes such as *GET4*, *KCTD5*, *NBPF9*, *SKT11* and *MTMR4*. While we didn't validate or further assess these genes and their ability to confer sensitivity to LuTate when expression is lost, they all provide potentially interesting targets. *STK11*, a tumour suppressor gene, has a well described role in the pathogenesis of cancer, and is the gene responsible for Peutz-Jeghers Syndrome 27. *KCTD5* has been identified as having a role in cell migration 28, 29; and GET4, as part of a larger protein complex with BAG6, has been shown to play a role in the recruitment of BRCA1 to sites of DNA damage 30 and promote tumour growth in models of colorectal cancer 31. Interestingly, *MTMR4* and *NBPF9* currently have no clear role in cancer or response to radiation.

**Figure 2:**
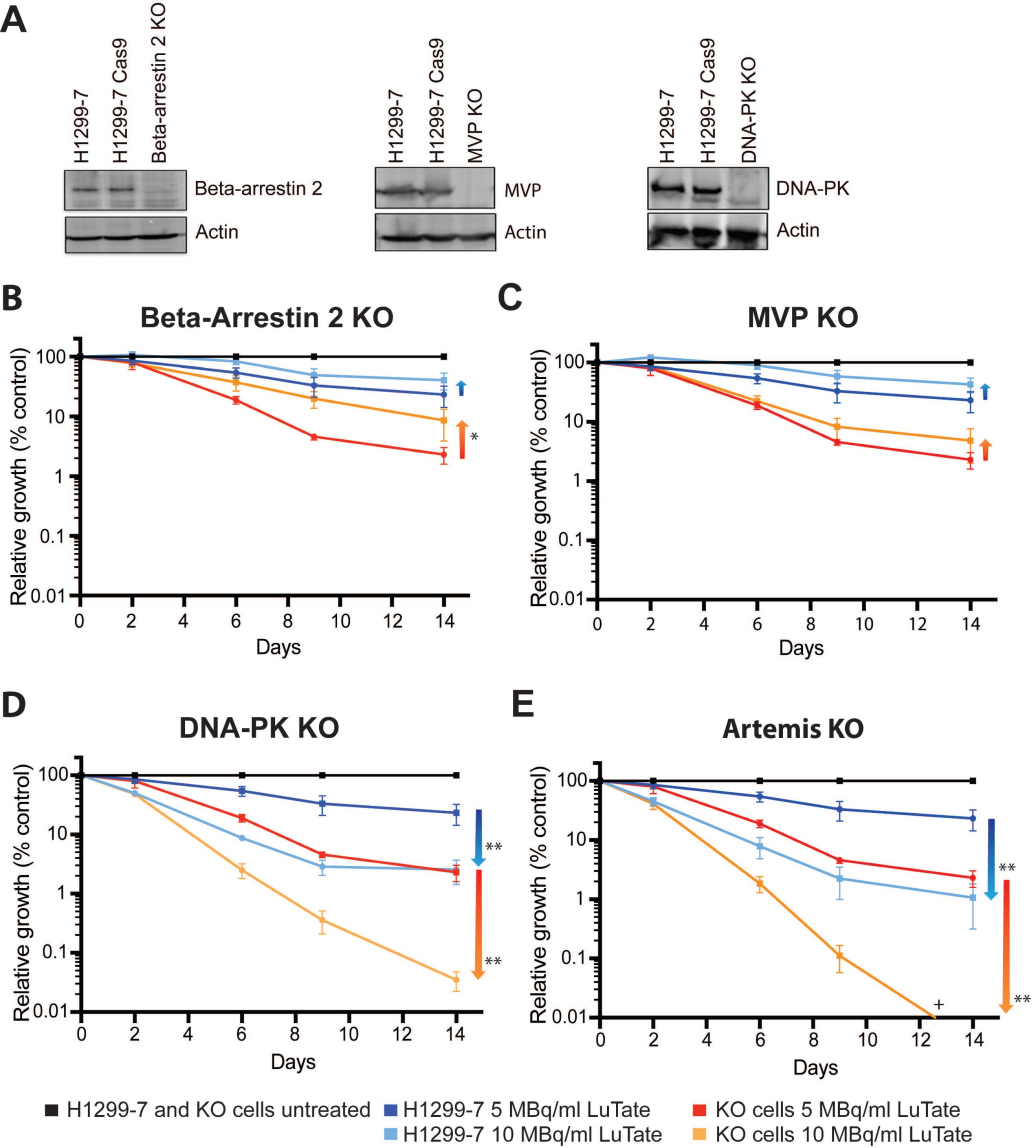
Validation of CRISPR screen resistance and sensitivity hits. **A)** Western blot showing loss of Beta-Arrestin 2, MVP and DNA-PK in the single-gene knockout cell lines generated for validation (H1299-7 cell lines Beta-Arrestin 2 KO, MVP KO, DNA-PK KO). Editing of *DCLRE1C*, (H1299-7 cell line Artemis KO) was confirmed through DNA sequencing. **B - E)** LuTate response curves, showing % relative growth, of the knockout cell lines, as compared to control H1299-7 cells. Cells were treated over four hours at 5 MBq/mL and 10 MBq/mL LuTate and growth tracked over 14 days. H1299-7 control response curves on all plots are untreated control in black, 5 MBq/mL LuTate in blue and 10 MBq/mL LuTate in red. Response curves on all plots of (B) Beta-Arrestin 2 KO, (C) MVP KO, (D) DNA-PK KO and (E) Artemis KO are untreated control in black, 5 MBq/mL LuTate in light blue and 10 MBq/mL LuTate in orange. Arrows indicate the direction of shift of response to LuTate of cells with KO, with either decreased response (resistance in the Beta-Arrestin 2 KO and MVP KO) or increased response to LuTate (sensitivity in the DNA-PK KO and Artemis KO). * p value <0.05, ** p value <0.005, + Artemis KO treated with 10 MBq/ml LuTate Day 14 value is 0.0038%.

Four candidate genes were chosen for validation of the CRISPR-Cas9 screen results, *ARRB2* and *MVP* from the resistance arm, and for sensitivity *PRKDC* (as the most significant gene) and *DCLRE1C* (coding for Artemis). Single-gene knockout cell lines were generated using CRISPR technology (using guides identified in the initial screen), creating the cells lines H1299-7 Beta-Arrestin 2 Knock-Out (KO), MVP KO, DNA-PK KO, and Artemis KO, with loss of protein expression in these cell lines confirmed by Western blot (Figure 2A and Figure S3A-C). Artemis protein expression could not be quantified accurately due to the unreliability of the several antibody detection methods tested, and so gene editing (a single base-pair deletion) was confirmed through DNA sequencing (Figure S4).

We assessed response to LuTate in the KO cell lines through CRISPR validation growth assays, tracking cell growth over a 14-day period. In the pooled genome-wide screen context, sub-populations are in competition and the effect of the gRNA/gene knockout is identified by relative enrichment/depletion compared to the original size of the sub-population. In the growth assays, parental and single-gene KO cell lines treated with different LuTate exposures were expanded individually and the changing sizes of the populations were compared as relative growth over time as a percentage of the control (illustrated in supplementary Figure S1). Initial studies at 5 MBq/mL and 10 MBq/mL LuTate confirmed that the control cell line H1299-7 and the Cas9 transduced cell line H1299-7 Cas9 responded similarly (Figure S5A). All four KO cell lines assessed in the growth assays exhibited responses to LuTate consistent with their identification within the screen (either resistance or sensitivity, Figure 2B-E), and showed increasing relative enrichment/depletion over time and with increasing LuTate exposure, confirming that the design of the pooled genome-wide screen was well calibrated to identify genes of interest in our chosen context.

### Loss of Beta-Arrestin 2 and MVP result in resistance to LuTate PRRT

Resistance to LuTate as a result of loss of Beta-Arrestin 2 (Figure 2B) and MVP (Figure 2C) in the H1299-7 cells was evident at both 5 MBq/mL and 10 MBq/mL LuTate. Both KO cell lines conferred a similar degree of resistance, with an approximate two-fold increase in the relative growth at 14 days after 5 MBq/mL. A stronger resistance phenotype was observed in the Beta-Arrestin 2 KO cells when treated at 10 MBq/mL, with a near four-fold increase in survival after 14 days (p-value 0.015). However, when the LuTate dose was increased to 20 MBq/mL expansion of the parental H1299-7 cells was reduced to <0.1% survival, which made it difficult to assess the conferral of resistance from the candidate gene knockouts (Figure S5B). These results highlighted the importance of carefully titrating the radionuclide dose in the pooled genome-wide screen.

**Figure 3:**
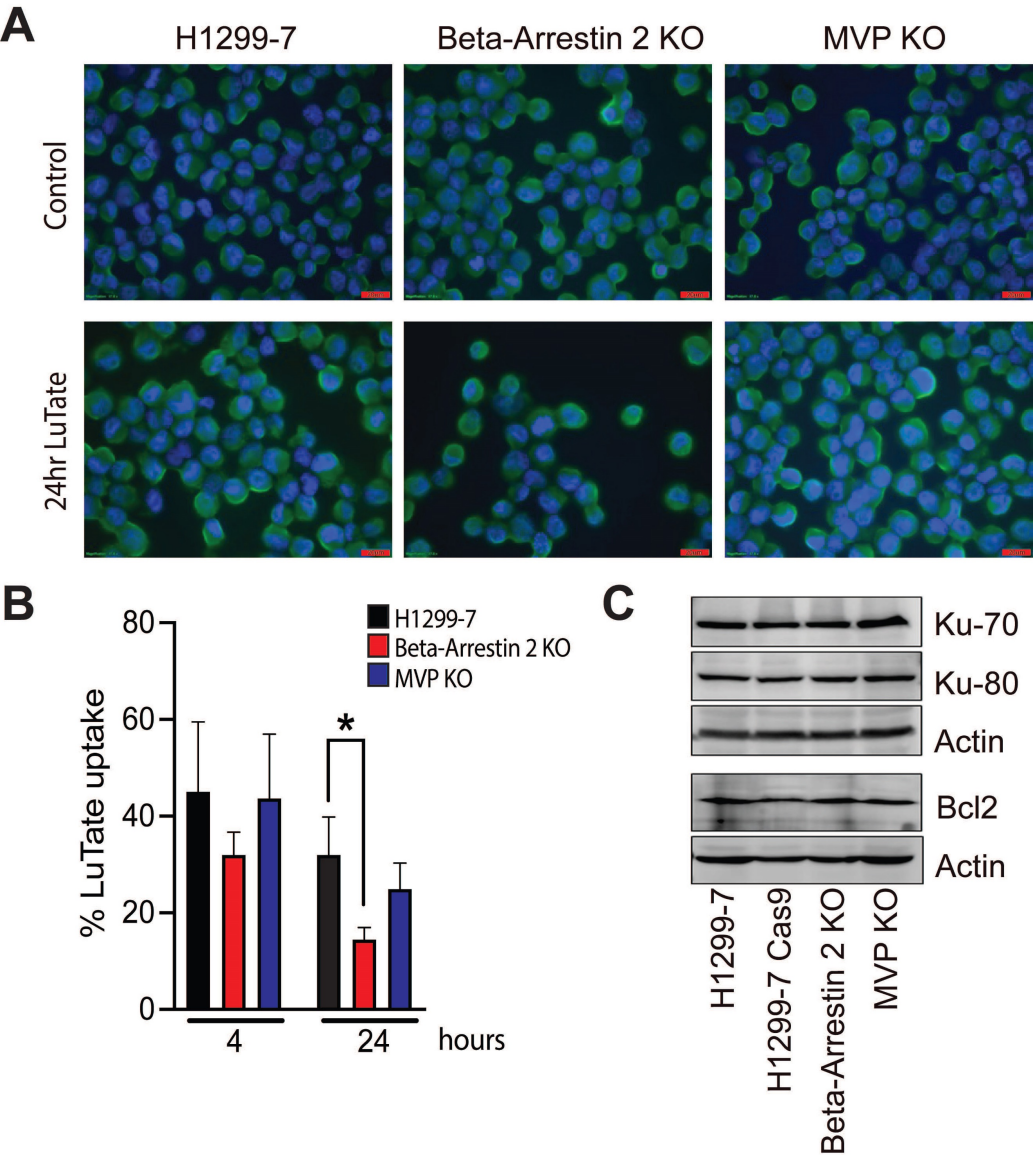
Assessment of mechanisms that may lead to the development of resistance to LuTate when Beta-Arrestin 2 and MVP are lost. **A)** SSTR2 expression by IF in H1299-7, Beta-Arrestin 2 KO, and MVP KO cells, both untreated and LuTate treated. Cells were treated with 5 MBq/mL LuTate for 4 h, then replated and collected for staining at 24 h post LuTate dose. Scale bar is 20 μm. **B)** LuTate retention assay of the H1299-7, Beta-Arrestin 2 KO, and MVP KO cells after treatment with 5 MBq/mL LuTate. Retention of LuTate was assessed at the end of the 4 h treatment period, and at 24 h post treatment (* p-value 0.01). **C)** Western blot of expression of Ku-70, Ku-80 and Bcl-2 in the control, MVP KO, and Beta-Arrestin 2 KO cell lines.

As seen in Figure 3A, SSTR2 expression and localisation was not substantially changed in the KO cell lines, with or without LuTate exposure. Interestingly after 24 h in culture in fresh medium, following 4 h of treatment with 5 MBq/mL LuTate, H1299-7 Beta-Arrestin 2 KO cells retained less LuTate than H1299-7 control cells (Figure 3B), with only 14.5% of LuTate retained, as compared to 32% in the control cells (p-value 0.01). There was no alteration to the amount of LuTate retained in the H1299-7 MVP KO cells. MVP has been identified as having a potential role in DNA-damage response pathways through interactions with Ku-70, Ku-80 and Bcl2. Knock-out of MVP in the H1299-7 cells had no effect on Ku70, Ku80 or Bcl2 expression (Figure 3C).

### Loss of DNA Damage response genes increases sensitivity to LuTate PRRT

From the sensitivity arm of the screen, loss of DNA-PK (Figure 2D) and Artemis (Figure 2E) both significantly sensitised H1299-7 cells to LuTate treatment. Consistent with the data from the screen, sensitisation of cells increased over time, and the degree to which cells were sensitised was markedly higher than the degree to which we could detect resistance. At Day 14, H1299-7 DNA-PK KO cells were 10 times more sensitive to LuTate than control cells (p value 0.0017), when treated at 5 MBq/mL. Loss of Artemis increased this further so that Artemis KO cells were 20 times more sensitive to LuTate than control cells (p value 0.0021). When LuTate treatment was increased to 10 MBq/mL, both DNA-PK KO and Artemis KO cells were almost completely eradicated by Day 14.

**Figure 4:**
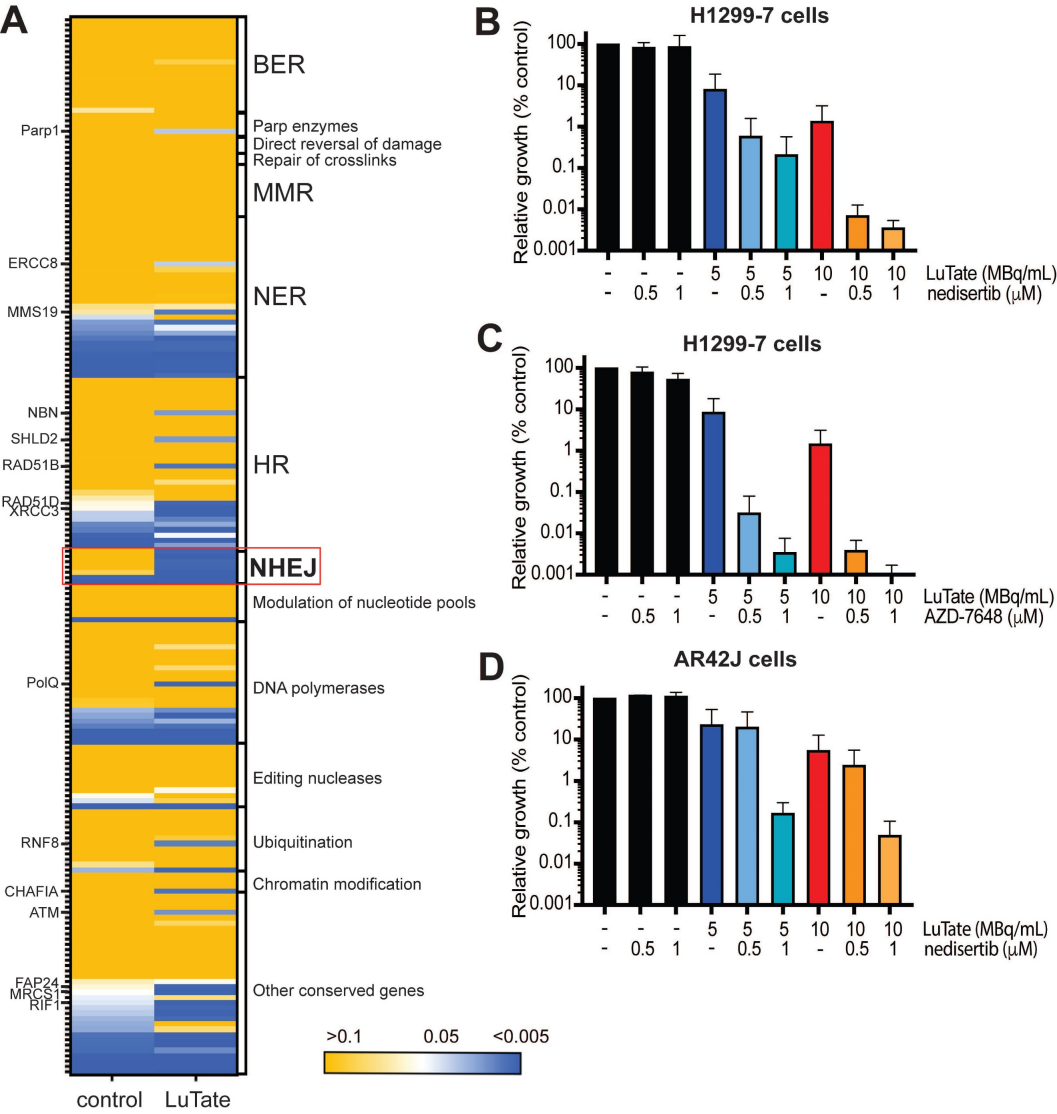
Alterations to DNA-damage repair pathways and role of DNA-PK inhibition in sensitivity to LuTate. **A)** Heat map of p-value change for DNA-damage repair pathway genes between control and LuTate treated cells in the sensitivity arm of the CRISPR screen. Genes are grouped by pathway and ranked within each group from least significant (yellow, p-value >0.05) to most significant (blue, p-value <0.005) by the p-value of the control group. **B and C)** Cell growth response at Day 14, shown as % relative growth as compared to control of H1299-7 cells treated with 0, 5 or 10 MBq/mL LuTate alone, or in combination with 0.5 μM or 1 μM of DNA-PK inhibitor nedisertib (B) or AZD-7648 (C). **D)** Cell growth response at Day 14, shown as % relative growth as compared to control of AR42J cells treated with 0, 5 or 10 MBq/mL LuTate alone, or in combination with 0.5 μM or 1 μM of DNA-PK inhibitor nedisertib.

Given that in the sensitivity arm of our screen many DDR genes were identified as being specifically depleted in LuTate-treated cells (according to our pre-established significance thresholds), we expanded our analysis - from the two genes validated - to assess the change in all DNA-damage pathway genes in response to LuTate (gene list taken from Lange et al, 32). In Figure 4A, we mapped the p-value (from the sensitivity arm of the screen) of DNA damage pathway genes in both untreated and LuTate treated Day 21 samples, with the genes classified by their canonical pathway involvement. Although individual genes across a variety of pathways showed a significant p-value in the LuTate treated samples (p-value <0.005, blue), while remaining unaltered in control samples (p-value >0.05, yellow), the dependence on the NHEJ pathway is clear. Pathways such as base excision repair (BER), which has been hypothesised as being a key pathway in response to PRRT, due to the predominance of SSB in response to β-emitting isotopes, shows no single genes as being important in the response to LuTate (all p-values in both control and treated groups were >0.05). Individual genes that show more significance in LuTate treated samples than controls, outside of those genes within the NHEJ pathway, include *POLQ*, *RAD51B*, *RAD51D*, *XRCC3*, *MRCS1* and *ATM*, several of which are involved in the HR pathway, though this pathway, as a whole, was not significantly altered.

### Inhibition of DNA-PK sensitises cells to LuTate PRRT *in vitro*

The focus of our study then turned to the most significant hit of the sensitivity arm of the screen, DNA-PK and assessed the impact of pharmacological inhibition of DNA-PK, utilising two inhibitors nedisertib (M3814 or peposertib) and AZD-7648 in combination with LuTate. Nedisertib is currently being tested in several clinical trials in combination with both radiotherapy and LuTate PRRT (Clinicaltrials.gov) while both nedisertib 33-37 and AZD-7648 38-40 have shown efficacy in combination with radiotherapy in pre-clinical models. Unfortunately, there were no Artemis inhibitors commercially available, and so the Artemis knockout studies could not be replicated using pharmacological inhibition.

In the H1299-7 cells, both DNA-PK inhibitors when combined with LuTate, resulted in sensitivity at Day 14 comparable to that observed with the H1299-7 DNA-PK KO cells (Figure 4B and 4C). The combination of 10 MBq/mL LuTate and either dose of nedisertib resulted in less than 0.01% of cells surviving to Day 14 (Figure 4B). While the combination of AZD-7648 and LuTate was more effective, with a similar level of cell survival observed when AZD-7648 was combined with 5 MBq/mL LuTate (Figure 4C). Both nedisertib and AZD-7648 are competitive inhibitors for the ATP-binding site of DNA-PKcs, and were similarly effective in the H1299-7 cells, nedisertib was chosen as the model DNA-PK inhibitor as it is further through clinical development. *In vitro* we utilised a second cell line that is routinely used for the study of LuTate PRRT, the rat pancreatic carcinoma cell line AR42J. In this cell line, we showed that the combination of LuTate and nedisertib resulted in increased sensitivity, albeit to a lesser extent that that observed in the H1299-7 cells (Figure 4D).

### Pre-clinical response to the combination of LuTate PRRT and DNA-PK inhibition

**Figure 5:**
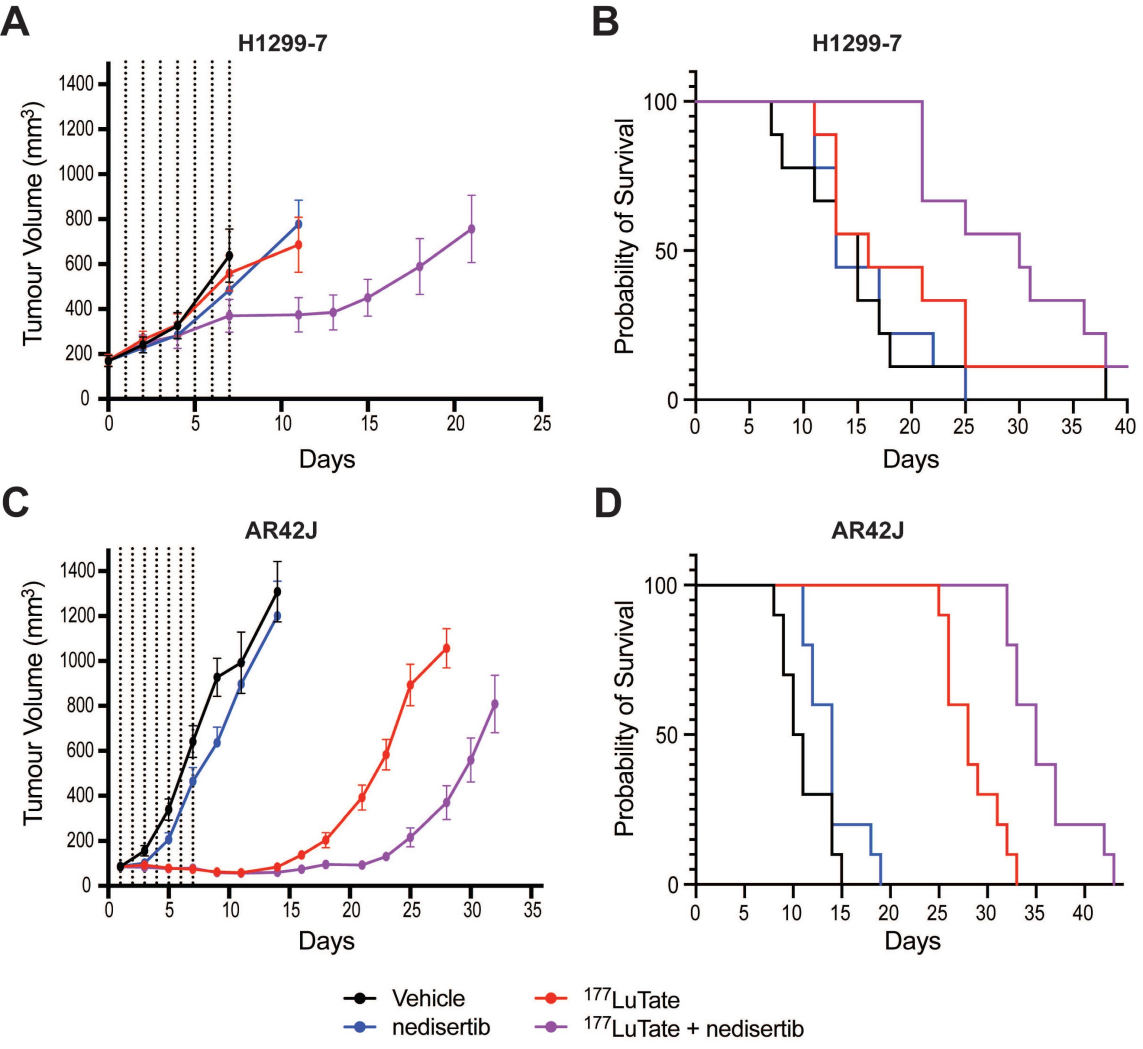
LuTate PRRT and nedisertib in combination results in tumour control and prolonged survival in *in vivo* models. **A)** H1299-7 cells were implanted into mice and treated with a single dose of 6 MBq LuTate with and without 6 days of nedisertib treatment (150 mg/kg). Tumour growth was monitored, and average tumour volume of 9-10 mice per group plotted. **B)** Survival curve of H1299-7 tumours treated with LuTate, nedisertib or combination. **C)** AR42J cells were implanted into mice and treated with a single dose of 9 MBq LuTate with and without 6 days of nedisertib treatment (150 mg/kg). Tumour growth was monitored, and average tumour volume of 9-10 mice per group plotted. **D)** Survival curve of AR42J tumours treated with LuTate, nedisertib or combination.

*In vivo* experiments with nedisertib showed its combination with LuTate to be well-tolerated (as measured by animal body weight during the experiments, Figure S6A and S6B) and improved survival in both the H1299-7 and AR42J models (Figure 5) even with the selection of administered activities of LuTate known to have little effect when given alone. H1299-7 and AR42J cells were grown as xenografts in mice, treated with a single sub-therapeutic dose of LuTate followed by 6 days of nedisertib. The first dose of nedisertib was given 24 h post the LuTate dose, with this timepoint chosen to avoid radiosensitisation of bone marrow cells prior to blood clearance of LuTate. H1299-7 xenografts treated with 6 MBq LuTate or nedisertib alone, showed no impact on tumour growth or survival as compared to untreated controls. The combination of LuTate and nedisertib resulted in tumour control for at least 7 days post the end of treatment (Figure 5A) and doubled the median survival to 30 days (survival curve p-value 0.024, Figure 5B).

In AR42J tumours, the LuTate dose was increased such that a more robust response to single agent treatment was observed, allowing us to assess the impact of the addition of nedisertib to an already effective dose of PRRT. In this model, 9 MBq of LuTate resulted in 10 days of tumour control post the end of treatment, with this increasing to 17 days with the addition of nedisertib (Figure 5C). Although activity of nedisertib against rat DNA-PK has not been published to our knowledge, the results are consistent with the effect seen in the human tumour xenograft model. Median survival of AR42J xenograft mice increased to 35 days when treated with the combination, up from 28 days when treated with LuTate alone, and 10 days in the vehicle control treated animals (p-value 0.0001, Figure 5D).

### The combination of LuTate PRRT and DNA-PK inhibition is effective in resistant cell lines

**Figure 6:**
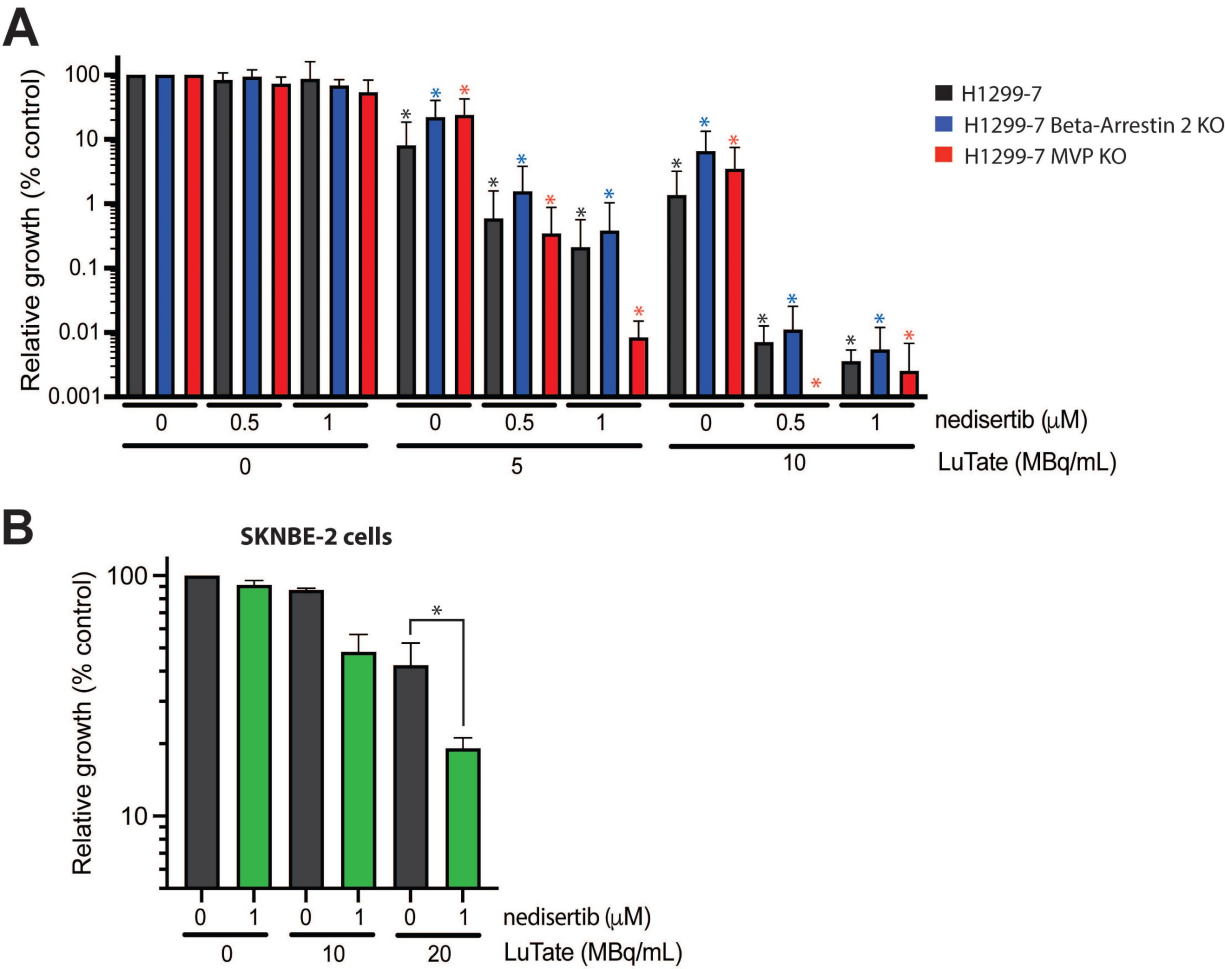
Assessing the impact of nedisertib in combination with LuTate in models of resistance to LuTate. **A)** Cell growth response at Day 14, shown as % relative growth as compared to control, of H1299-7, Beta-Arrestin 2 KO and MVP KO cell lines treated with 0, 5 or 10 MBq/mL LuTate alone, or in combination with 0.5 μM or 1 μM of DNA-PK inhibitor nedisertib. (* p-value <0.05 as compared to control) **B)** Cell growth response at Day 14, shown as % relative growth as compared to control, of SKNBE-2 cells treated with 0, 10 or 20 MBq/mL LuTate alone, or in combination with 1 μM of DNA-PK inhibitor nedisertib (* p-value <0.05).

Returning to the problem of resistance, we wanted to determine whether the addition of nedisertib to LuTate could provide a promising avenue for overcoming resistance. To do this, we utilised the H1299-7 Beta-Arrestin 2 KO and MVP KO cell lines generated in this study, and a third human cell line, SKNBE-2, a neuroblastoma cell line with reduced SSTR2 expression and limited response to LuTate 19. The combination of nedisertib and LuTate on both the H1299-7 Beta-Arrestin 2 KO and MVP KO cells was effective at increasing sensitivity to LuTate in both cell lines *in vitro* (Figure 6A). This was particularly evident in the cells with loss of MVP, where the combination seemed most effective, with almost complete eradication of MVP KO cells observed at Day 14 after treatment with 5 MBq/mL and 1 µM nedisertib. SKNBE-2 cells are more resistant to LuTate than the other cell lines used in this study, with doses of less than 10 MBq/mL having little effect, and 20 MBq/mL reducing survival by only 50%. In combination, 20 MBq/mL LuTate and 1 µM nedisertib resulted in less than 20% of cells surviving at Day 14 post treatment (Figure 6B).

## Discussion

Multiple factors contribute to the variable response of advanced NET to PRRT. Intra-tumoural factors such as proliferation rate 3 and the presence of hypoxia 41 are associated with either increased or decreased objective response rates, respectively. The level of SSTR2 expression (measured by the Krenning score), location of the primary tumour and size of tumour deposits have also been shown to impact on response to PRRT 42, 43. However, even in NET arising from the same primary site, and of similar grade and Krenning score, highly variable responses are observed clinically. Furthering the understanding of underlying genetic variations that play a role in response to PRRT could provide insight into how to better manage NET patients. Resistance to LuTate PRRT treatment in the clinic has proved especially challenging, with no approved biomarkers able to predict the development of resistance, although a transcriptomic approach (NETest) has been described 44.

To start to address these questions we undertook a genome-wide CRISPR screen in the context of PRRT treatment, the validation of which required significant optimisation and customisation to the unique context of radionuclide therapy *in vitro*. If the cell survival after LuTate treatment is too high, only gene knockouts that produce extreme sensitivity will result in detectable depletion. Equally, if LuTate treatment results in minimal cell survival, many gene knockouts will be depleted despite the gene loss having little-to-no effect. If depletion and enrichment was assessed too early after the LuTate treatment, only gene knockouts which modify immediate cell survival would be identified. Yet, allowing time for additional sensitivity/resistance mechanisms (e.g. changes in growth rates, senescence etc.) to alter the longer-term expansion of a knockout sub-population, and comparisons across time, required sub-culturing a very large population of cells over several weeks including removing large samples of cells for analysis at each timepoint. Lastly, if the sub-population of cells carrying a given gRNA is too low at the start of the screen, or at the time of each harvest/sub-culture, it can become lost from the population by chance resulting in significant false-positive results, hence requiring maintenance of at least 500-fold coverage per gRNA throughout the assay. Together, these factors shaped the parameters of how the genome-wide CRISPR-Cas9 screens and the subsequent validation growth assays were designed.

Cell survival/reproductive potential in the context of radiation exposure would classically be determined using clonogenic assays, normalised from variable numbers of cells plated and the inherent cloning efficiency of the cells 45-47. However, the H1299-7 cells did not show a robust and consistent plating efficiency and so required us to develop the CRISPR validation growth assay, which ultimately had several advantages. Firstly, the growth assay gave the ability to follow population growth changes over time; a key requirement as the cells continue to be irradiated throughout the assay. It also incorporated the relevant mechanisms that could have produced the enrichment/depletion in the original screen, rather than focusing on reproductive potential alone. The growth assay design was resistant to the variable dose deposition that would have occurred from cross-fire with re-plating cells at different densities from sparse to highly clustered. Furthermore, it closely replicated the conditions of the original screen, which was not performed under colony-forming conditions. The identification of candidates which were successfully validated, and the identification of candidates within biological pathways with functional links to radionuclide-induced DNA damage as well as novel candidates, suggests that the experimental design was well calibrated in terms of parameter selection and statistical thresholds.

In our study, we identified several genes that when lost result in resistance to LuTate, including *ARRB2* and* MVP*. Beta-Arrestin 2 (*ARRB2*) through its role as a scaffolding protein involved in the localisation, regulation of activity and recycling of G-protein coupled receptors, including SSTR2 48, 49, provides an indirect mechanism of regulation of SSTR2 and therefore LuTate binding. The retention of LuTate in our assay was a functional measurement incorporating LuTate binding, internalisation and export, the recycling of SSTR2 receptors to allow re-binding, and any other mechanisms that alter the actual exposure to LuTate. The demonstration of reduced LuTate binding in cells with Beta-Arrestin 2 KO likely contributes to the increased cell survival compared to the parental cells and fits with its known role in modulating SSTR2. This provides a model of how patients with high Krenning scores (high SSTR2) may not respond as well as expected. Clinically, decreased radioligand retention is likely to reduce cumulative activity in tumour lesions and would thereby reduce the radiation dose received. Identifying this mechanism of resistance could potentially be achieved by imaging with tracers that can be imaged out to later timepoints, like ^64^Cu-MecoSAR-octreotate (CuSARTATE), as this may provide for more accurate dosimetry estimation in a setting of reduced retention 50.

The second gene identified, *MVP*, (also known as LRP, human lung resistance protein) is the major component of ribonucleoprotein structures, called vaults, thought to play a role in multi-drug resistance phenotypes and cellular transport 51, 52. Additionally, MVP has been identified as involved in the regulation of DDR pathways, specifically the NHEJ and HR pathways, through interactions that result in modulation of expression of the DNA-PK binding proteins Ku70 and Ku80 52, 53 and in cell survival where Bcl2 expression was reduced with the loss of MVP expression 54. In our study MVP KO had no effect on Ku70, Ku80 or Bcl2 expression in the H1299-7 cells, suggesting that modulation of these known MVP interaction partners was not an indirect PRRT resistance mechanism.

The LuTate resistance mechanism of MVP is more complicated to tease apart, due in part to its role in the DDR, and particularly the NHEJ pathway, which our screen identified as strongly involved in sensitivity to LuTate. Also contradicting our data, several studies involving loss of MVP and radiation therapy have suggested the development of radiosensitivity 52, 55-58, as opposed to the radioresistance we observed. Indeed, in patient cohorts, increased MVP expression has been strongly associated with local disease-free survival and radiation resistance in patients with squamous cell carcinoma of the oropharynx who were treated with radiotherapy 55, 56. And, in cervical cancer, the overexpression of MVP was strongly correlated with radioresistance, while a cohort of patients with low MVP expression showed excellent survival rates 57, 58. As such the role of MVP in PRRT resistance requires further consideration, though our study suggests that modulating the cells' ability to respond to the DNA damage inflicted by PRRT may provide answers not only to the question of increasing efficiency of the treatment, but also to understanding the development of resistance.

The role of the DNA-damage response in regulating cellular sensitivity to PRRT is well known. In our previous work, we showed that the inhibition of PARP - using the inhibitor talazoparib in combination with LuTate - enhanced cell death and increased survival in *in vitro* and *in vivo* models 19. While both DNA-PK inhibitors used in this study, nedisertib and AZD-7648, have previously been shown to increase response when used in combination with external beam radiotherapy 33-37, we have shown enhanced efficacy of LuTate PRRT, *in vitro* and *in vivo*, when combined with the inhibition of DNA-PK, as was also observed in a recent paper by Reuvers et al 59. Additionally, we have shown that the combination of nedisertib and LuTate *in vivo*, was effective even when the dose of LuTate had little effect alone.

Our results suggest an opportunity for the clinical translation of this treatment combination. To this end, several clinical trials are evaluating the tolerability and efficacy of nedisertib in humans. A phase I clinical trial showed that nedisertib was well tolerated but showed only modest efficacy in unselected tumours 60. Current clinical trials are focusing on the combination of nedisertib and radiotherapy in the setting of pancreatic cancers (NCT04172532) and glioblastoma and gliosarcoma (NCT04555577), as well as others. Additionally, there are two studies investigating the combination of nedisertib and radionuclide therapy, one utilising radium-223 dichloride in the setting of castrate resistant prostate cancer (NCT04071236), and a Phase 1b trial assessing nedisertib and LuTate PRRT in pancreatic neuroendocrine tumours (NCT04750954).

Of particular interest in our study is evidence that the combination of DNA-PK inhibitors and LuTate showed a level of effectiveness within *in vitro* models that displayed resistance to LuTate. As such, the clinical use of PRRT in combination with a DNA-PK inhibitor may prove particularly useful in patients with previously identified resistance or relatively low Krenning scores. Thus, our study provides further evidence that DDR inhibitors, particularly DNA-PK inhibitors, could provide promising avenues to improving response to PRRT in NET patients.

## Supplementary Material

Supplementary figures and tables 1-3.

Supplementary table 4.

## Figures and Tables

**Table 1:** PCR setup

Reagent	Amount (μl)
10x Ex-Taq reaction buffer	10
2.5 mM dNTP	8
100 μM P5 primer mix	0.5
100 μM P7 primer	10
genomic DNA (1 μg/μl)	10
Ex-Taq polymerase (250 U, 5 U/μl)	0.75
H_2_O	up to 100

## Abbreviations

NET: neuroendocrine tumours
PRRT: peptide receptor radionuclide therapy
LuTate: ^177^Lutetium- DOTA-octreotate
SSTR2: somatostatin type 2 receptor
DDR: DNA-damage repair
DSB: double strand DNA break
SSB: single strand DNA break
HR: homologous recombination
NHEJ: non-homologous end-Joining
BER: base-excision repair
MOI: multiplicity of infection
KO: knockout

## References

[1] Strosberg J, El-Haddad G, Wolin E, Hendifar A, Yao J, Chasen B (2017). Phase 3 Trial of (177)Lu-Dotatate for Midgut Neuroendocrine Tumors. N Engl J Med.

[2] Hicks RJ, Kwekkeboom DJ, Krenning E, Bodei L, Grozinsky-Glasberg S, Arnold R (2017). ENETS Consensus Guidelines for the Standards of Care in Neuroendocrine Neoplasia: Peptide Receptor Radionuclide Therapy with Radiolabeled Somatostatin Analogues. Neuroendocrinology.

[3] Sorbye H, Kong G, Grozinsky-Glasberg S (2020). PRRT in high-grade gastroenteropancreatic neuroendocrine neoplasms (WHO G3). Endocr Relat Cancer.

[4] Kong G, Grozinsky-Glasberg S, Hofman M, Callahan J, Meirovitz A, Maimon O (2017). Efficacy of Peptide Receptor Radionuclide Therapy (PRRT) for Functional Metastatic Paraganglioma and Phaeochromocytoma. J Clin Endocrinol Metab.

[5] Carrasquillo JA, Chen CC, Jha A, Pacak K, Pryma DA, Lin FI (2021). Systemic Radiopharmaceutical Therapy of Pheochromocytoma and Paraganglioma. J Nucl Med.

[6] Jha A, Taïeb D, Carrasquillo JA, Pryma DA, Patel M, Millo C (2021). High-Specific-Activity-^131^I-MIBG vs ^177^Lu-DOTATATE based peptide receptor radionuclide therapy: an evolving conundrum in targeted radionuclide therapy of metastatic pheochromocytoma and paraganglioma. Clin Cancer Res.

[7] Kong G, Hofman MS, Murray WK, Wilson S, Wood P, Downie P (2016). Initial Experience With Gallium-68 DOTA-Octreotate PET/CT and Peptide Receptor Radionuclide Therapy for Pediatric Patients With Refractory Metastatic Neuroblastoma. J Pediatr Hematol Oncol.

[8] Brabander T, van der Zwan WA, Teunissen JJM, Kam BLR, Feelders RA, de Herder WW (2017). Long-Term Efficacy, Survival, and Safety of [(177)Lu-DOTA(0),Tyr(3)]octreotate in Patients with Gastroenteropancreatic and Bronchial Neuroendocrine Tumors. Clin Cancer Res.

[9] Strosberg J, Leeuwenkamp O, Siddiqui MK (2021). Peptide receptor radiotherapy re-treatment in patients with progressive neuroendocrine tumors: A systematic review and meta-analysis. Cancer Treat Rev.

[10] Del Prete M, Buteau FA, Arsenault F, Saighi N, Bouchard LO, Beaulieu A (2019). Personalized (177)Lu-octreotate peptide receptor radionuclide therapy of neuroendocrine tumours: initial results from the P-PRRT trial. Eur J Nucl Med Mol Imaging.

[11] Claringbold PG, Brayshaw PA, Price RA, Turner JH (2011). Phase II study of radiopeptide 177Lu-octreotate and capecitabine therapy of progressive disseminated neuroendocrine tumours. Eur J Nucl Med Mol Imaging.

[12] Hubble D, Kong G, Michael M, Johnson V, Ramdave S, Hicks RJ (2010). 177Lu-octreotate, alone or with radiosensitising chemotherapy, is safe in neuroendocrine tumour patients previously treated with high-activity 111In-octreotide. Eur J Nucl Med Mol Imaging.

[13] Lewin J, Cullinane C, Akhurst T, Waldeck K, Watkins DN, Rao A (2015). Peptide receptor chemoradionuclide therapy in small cell carcinoma: from bench to bedside. Eur J Nucl Med Mol Imaging.

[14] McLaughlin M, Patin EC, Pedersen M, Wilkins A, Dillon MT, Melcher AA (2020). Inflammatory microenvironment remodelling by tumour cells after radiotherapy. Nat Rev Cancer.

[15] Brown JS, O'Carrigan B, Jackson SP, Yap TA (2017). Targeting DNA Repair in Cancer: Beyond PARP Inhibitors. Cancer Discov.

[16] Chatterjee N, Walker GC (2017). Mechanisms of DNA damage, repair, and mutagenesis. Environ Mol Mutagen.

[17] Purohit NK, Shah RG, Adant S, Hoepfner M, Shah GM, Beauregard JM (2018). Potentiation of (177)Lu-octreotate peptide receptor radionuclide therapy of human neuroendocrine tumor cells by PARP inhibitor. Oncotarget.

[18] Nonnekens J, van Kranenburg M, Beerens CE, Suker M, Doukas M, van Eijck CH (2016). Potentiation of Peptide Receptor Radionuclide Therapy by the PARP Inhibitor Olaparib. Theranostics.

[19] Cullinane C, Waldeck K, Kirby L, Rogers BE, Eu P, Tothill RW (2020). Enhancing the anti-tumour activity of (177)Lu-DOTA-octreotate radionuclide therapy in somatostatin receptor-2 expressing tumour models by targeting PARP. Sci Rep.

[20] Doench JG, Hartenian E, Graham DB, Tothova Z, Hegde M, Smith I (2014). Rational design of highly active sgRNAs for CRISPR-Cas9-mediated gene inactivation. Nat Biotechnol.

[21] Doench JG, Fusi N, Sullender M, Hegde M, Vaimberg EW, Donovan KF (2016). Optimized sgRNA design to maximize activity and minimize off-target effects of CRISPR-Cas9. Nat Biotechnol.

[22] Li W, Xu H, Xiao T, Cong L, Love MI, Zhang F (2014). MAGeCK enables robust identification of essential genes from genome-scale CRISPR/Cas9 knockout screens. Genome Biol.

[23] Iyer VS, Jiang L, Shen Y, Boddul SV, Panda SK, Kasza Z (2020). Designing custom CRISPR libraries for hypothesis-driven drug target discovery. Comput Struct Biotechnol J.

[24] Doench JG (2018). Am I ready for CRISPR? A user's guide to genetic screens. Nat Rev Genet.

[25] Otten ABC, Sun BK (2020). Research Techniques Made Simple: CRISPR Genetic Screens. J Invest Dermatol.

[26] Parry JJ, Eiblmaier M, Andrews R, Meyer LA, Higashikubo R, Anderson CJ (2007). Characterization of somatostatin receptor subtype 2 expression in stably transfected A-427 human cancer cells. Mol Imaging.

[27] Bourouh M, Marignani PA (2022). The Tumor Suppressor Kinase LKB1: Metabolic Nexus. Front Cell Dev Biol.

[28] Canales J, Cruz P, Diaz N, Riquelme D, Leiva-Salcedo E, Cerda O (2020). K(+) Channel Tetramerization Domain 5 (KCTD5) Protein Regulates Cell Migration, Focal Adhesion Dynamics and Spreading through Modulation of Ca(2+) Signaling and Rac1 Activity. Cells.

[29] Rivas J, Diaz N, Silva I, Morales D, Lavanderos B, Alvarez A (2020). KCTD5, a novel TRPM4-regulatory protein required for cell migration as a new predictor for breast cancer prognosis. FASEB J.

[30] Krenciute G, Liu S, Yucer N, Shi Y, Ortiz P, Liu Q (2013). Nuclear BAG6-UBL4A-GET4 complex mediates DNA damage signaling and cell death. J Biol Chem.

[31] Koike K, Masuda T, Sato K, Fujii A, Wakiyama H, Tobo T (2022). GET4 is a novel driver gene in colorectal cancer that regulates the localization of BAG6, a nucleocytoplasmic shuttling protein. Cancer Sci.

[32] Lange SS, Takata K, Wood RD (2011). DNA polymerases and cancer. Nat Rev Cancer.

[33] Gordhandas SB, Manning-Geist B, Henson C, Iyer G, Gardner GJ, Sonoda Y (2022). Pre-clinical activity of the oral DNA-PK inhibitor, peposertib (M3814), combined with radiation in xenograft models of cervical cancer. Sci Rep.

[34] Smithson M, Irwin RK, Williams G, McLeod MC, Choi EK, Ganguly A (2022). Inhibition of DNA-PK may improve response to neoadjuvant chemoradiotherapy in rectal cancer. Neoplasia.

[35] Sun Q, Guo Y, Liu X, Czauderna F, Carr MI, Zenke FT (2019). Therapeutic Implications of p53 Status on Cancer Cell Fate Following Exposure to Ionizing Radiation and the DNA-PK Inhibitor M3814. Mol Cancer Res.

[36] Wang W, McMillan MT, Zhao X, Wang Z, Jiang L, Karnak D (2022). DNA-PK Inhibition and Radiation Promote Antitumoral Immunity through RNA Polymerase III in Pancreatic Cancer. Mol Cancer Res.

[37] Zenke FT, Zimmermann A, Sirrenberg C, Dahmen H, Kirkin V, Pehl U (2020). Pharmacologic Inhibitor of DNA-PK, M3814, Potentiates Radiotherapy and Regresses Human Tumors in Mouse Models. Mol Cancer Ther.

[38] Fok JHL, Ramos-Montoya A, Vazquez-Chantada M, Wijnhoven PWG, Follia V, James N (2019). AZD7648 is a potent and selective DNA-PK inhibitor that enhances radiation, chemotherapy and olaparib activity. Nat Commun.

[39] Hong CR, Buckley CD, Wong WW, Anekal PV, Dickson BD, Bogle G (2022). Radiosensitisation of SCCVII tumours and normal tissues in mice by the DNA-dependent protein kinase inhibitor AZD7648. Radiother Oncol.

[40] Nakamura K, Karmokar A, Farrington PM, James NH, Ramos-Montoya A, Bickerton SJ (2021). Inhibition of DNA-PK with AZD7648 Sensitizes Tumor Cells to Radiotherapy and Induces Type I IFN-Dependent Durable Tumor Control. Clin Cancer Res.

[41] Bouleftour W, Rowinski E, Louati S, Sotton S, Wozny AS, Moreno-Acosta P (2021). A Review of the Role of Hypoxia in Radioresistance in Cancer Therapy. Med Sci Monit.

[42] Kong G, Grozinsky-Glasberg S, Hofman MS, Akhurst T, Meirovitz A, Maimon O (2019). Highly favourable outcomes with peptide receptor radionuclide therapy (PRRT) for metastatic rectal neuroendocrine neoplasia (NEN). Eur J Nucl Med Mol Imaging.

[43] Kong G, Thompson M, Collins M, Herschtal A, Hofman MS, Johnston V (2014). Assessment of predictors of response and long-term survival of patients with neuroendocrine tumour treated with peptide receptor chemoradionuclide therapy (PRCRT). Eur J Nucl Med Mol Imaging.

[44] Bodei L, Raj N, Do RK, Mauguen A, Krebs S, Reidy-Lagunes D (2023). Interim Analysis of a Prospective Validation of 2 Blood-Based Genomic Assessments (PPQ and NETest) to Determine the Clinical Efficacy of (177)Lu-DOTATATE in Neuroendocrine Tumors. J Nucl Med.

[45] Brix N, Samaga D, Hennel R, Gehr K, Zitzelsberger H, Lauber K (2020). The clonogenic assay: robustness of plating efficiency-based analysis is strongly compromised by cellular cooperation. Radiat Oncol.

[46] Franken NA, Rodermond HM, Stap J, Haveman J, van Bree C (2006). Clonogenic assay of cells *in vitro*. Nat Protoc.

[47] Pomp J, Wike JL, Ouwerkerk IJ, Hoogstraten C, Davelaar J, Schrier PI (1996). Cell density dependent plating efficiency affects outcome and interpretation of colony forming assays. Radiother Oncol.

[48] Matthees ESF, Haider RS, Hoffmann C, Drube J (2021). Differential Regulation of GPCRs-Are GRK Expression Levels the Key?. Front Cell Dev Biol.

[49] Peterson YK, Luttrell LM (2017). The Diverse Roles of Arrestin Scaffolds in G Protein-Coupled Receptor Signaling. Pharmacol Rev.

[50] Hicks RJ, Jackson P, Kong G, Ware RE, Hofman MS, Pattison DA (2019). (64)Cu-SARTATE PET Imaging of Patients with Neuroendocrine Tumors Demonstrates High Tumor Uptake and Retention, Potentially Allowing Prospective Dosimetry for Peptide Receptor Radionuclide Therapy. J Nucl Med.

[51] Berger W, Steiner E, Grusch M, Elbling L, Micksche M (2009). Vaults and the major vault protein: novel roles in signal pathway regulation and immunity. Cell Mol Life Sci.

[52] Lara PC, Pruschy M, Zimmermann M, Henriquez-Hernandez LA (2011). MVP and vaults: a role in the radiation response. Radiat Oncol.

[53] Lloret M, Lara PC, Bordon E, Fontes F, Rey A, Pinar B (2009). Major vault protein may affect nonhomologous end-joining repair and apoptosis through Ku70/80 and bax downregulation in cervical carcinoma tumors. Int J Radiat Oncol Biol Phys.

[54] Ryu SJ, An HJ, Oh YS, Choi HR, Ha MK, Park SC (2008). On the role of major vault protein in the resistance of senescent human diploid fibroblasts to apoptosis. Cell Death Differ.

[55] Henriquez-Hernandez LA, Moreno M, Rey A, Lloret M, Lara PC (2012). MVP expression in the prediction of clinical outcome of locally advanced oral squamous cell carcinoma patients treated with radiotherapy. Radiat Oncol.

[56] Silva P, West CM, Slevin N, Valentine H, Ryder WD, Hampson L (2007). Tumor expression of major vault protein is an adverse prognostic factor for radiotherapy outcome in oropharyngeal carcinoma. Int J Radiat Oncol Biol Phys.

[57] Henriquez-Hernandez LA, Lloret M, Pinar B, Bordon E, Rey A, Lubrano A (2011). BCL-2, in combination with MVP and IGF-1R expression, improves prediction of clinical outcome in complete response cervical carcinoma patients treated by radiochemotherapy. Gynecol Oncol.

[58] Lloret M, Lara PC, Bordon E, Rey A, Falcon O, Apolinario RM (2008). MVP expression is related to IGF1-R in cervical carcinoma patients treated by radiochemotherapy. Gynecol Oncol.

[59] Reuvers TGA, Verkaik NS, Stuurman D, de Ridder C, Groningen MCC, de Blois E (2023). DNA-PKcs inhibitors sensitize neuroendocrine tumor cells to peptide receptor radionuclide therapy *in vitro* and *in vivo*. Theranostics.

[60] van Bussel MTJ, Awada A, de Jonge MJA, Mau-Sorensen M, Nielsen D, Schoffski P (2021). A first-in-man phase 1 study of the DNA-dependent protein kinase inhibitor peposertib (formerly M3814) in patients with advanced solid tumours. Br J Cancer.

