# Supplementary material for: A genome-wide CRISPR/Cas9 screen identifies DNA-PK as a sensitiser to 177Lutetium-DOTA-octreotate radionuclide therapy
Source: Theranostics. 2023 Aug 28;13(14):4745–61. doi: 10.7150/thno.84628 (PMC10526672; doi:10.7150/thno.84628)
Supplement: Supplementary file 1 — Supplementary figures and tables 1-3. [file thnov13p4745s1.pdf]

Supplementary Figure 1. A) Diagrammatic representation of the growth assay, showing the splitting and replating process. Only one flask (or well of a plate) is maintained throughout the assay, with the cells from that well counted at each timepoint and then replated at a known concentration. In this representative figure the cells were replated at 50% of what was present in the well when counted. While in the assays, especially within the control groups, it could be as little as 10% of the counted population is replated, to maintain the same known concentration across the experiment at each timepoint. **B)** Calculations then extrapolated the total population cell number as if all cells had been replated at each time point, as in this example of the H1299-7 untreated control cells and the H1299-7 cells treated with 10 MBq/mL LuTate. This calculation was based on knowing the concentration of cells plated, and determining how many flasks would have been required were the whole population replated at that concentration. It was not technically possible to replate all cells at all timepoints due to the high number of flasks (or wells) that would be required. **C)** Normalisation of the data was performed to show the relative growth of treated cultures as compared to the control untreated cells (designated at 100% growth).

Supplementary Figure 2. A) FACS plot of GFP expression in Cas9 transduced H1299-7 cells. H1299-7 Cas9 cells were transduced with the GFP reporter vector, with loss of GFP expression determining efficacy of Cas9 activity. **B)** H1299-7 Cas9 cell growth over the course of the 21 days of the CRISPR screen. Cells were plated and 24 h later treated for four hours with either control, 0.5 MBq/mL or 2.5 MBq/mL LuTate and growth tracked over 21 days. **C)** H1299-7 Cas9 cell growth. Given the large number of cells required to attain 500-fold representation of individual gRNAs throughout the duration of the screen, the cell number was optimised to involve exposing the cells to LuTate in suspension before removal of unbound LuTate and replating. Cells were treated in suspension for four hours at 5 MBq/mL, at concentrations of 4×10^6 cells/mL, 8×10^6 cells/mL and 15×10^6 cells/mL in 10 mL (with actual cell numbers of 40×10^6, 80×10^6 and 150×10^6 cells). Cells were plated after treatment and growth tracked.

Supplementary Figure 3. Western blot analysis of the **(A)** H1299-7 Beta-Arrestin 2 KO, **(B)** H1299-7 MVP KO and **(C)** H1299-7 DNA-PK KO cells, showing loss of total protein.

Supplementary Figure 4. DNA sequence alignment of the Artemis knockout cell line (H1299-7 Artemis KO), as compared to H1299-7 cells. Alignment shows the presence of a single base pair deletion within the location (position 17) of the CRISPR gRNA sequence in the H1299-7 Artemis KO cells. This deletion has resulted in a frameshift mutation within the DNA sequence.

Supplementary Figure 5. A) LuTate growth assays, showing relative growth (as a % of control), of the H1299-7 Cas9 cells, as compared to control H1299-7 cells, treated at 5 MBq/mL and 10 MBq/mL LuTate over 14 days. Black - H1299-7 and H1299-7 Cas9 control, 5 MBq/mL LuTate on H1299-7 cells (blue), 5 MBq/mL LuTate on H1299-7 Cas9 cells (light blue), 10 MBq/mL LuTate on H1299-7 cells (red), and 10 MBq/mL LuTate on H1299-7 Cas9 cells (orange). **B)** LuTate growth assays, showing relative growth (as a % of control), of the H1299-7, Beta-Arrestin 2 KO, MVP KO, DNA-PK KO, and Artemis KO cells lines in response to treatment with 20 MBq/mL LuTate, in comparison to controls (+ the value of the Day 14 Artemis KO 20 MBq/mL data point is 0.000296 %, the axis was not extended to view this).

Supplementary Figure 6. The combination of nedisertib and LuTate was well-tolerated in both the H1299-7 tumours **(A)** and the AR42J tumours **(B)**, as demonstrated by monitoring of body weight change in the mice over the course of the experiment. Percent body weight change is shown as the % alteration in body weight as compared to the weight of the animals (designated as 100%) at the day of first treatment.

Supplementary Table 1. P5 and P7 primer sequences used in the Illuminia sequencing of the DNA samples from the CRISPR screen.

Supplementary Table 2. Guide RNA sequences used for the generation of the single gene knockout cells lines, Beta-Arrestin 2 KO, MVP KO, DNA-PK KO, and Artemis KO in the H1299-7 cells.

Supplementary Table 3. PCR primers used for sequencing of the H1299-7 Artemis KO cell line to confirm gene-editing as a result of CRISPR knockout.

Supplementary Table 4. Full data of the CRISPR screen

Supplementary Figure S1

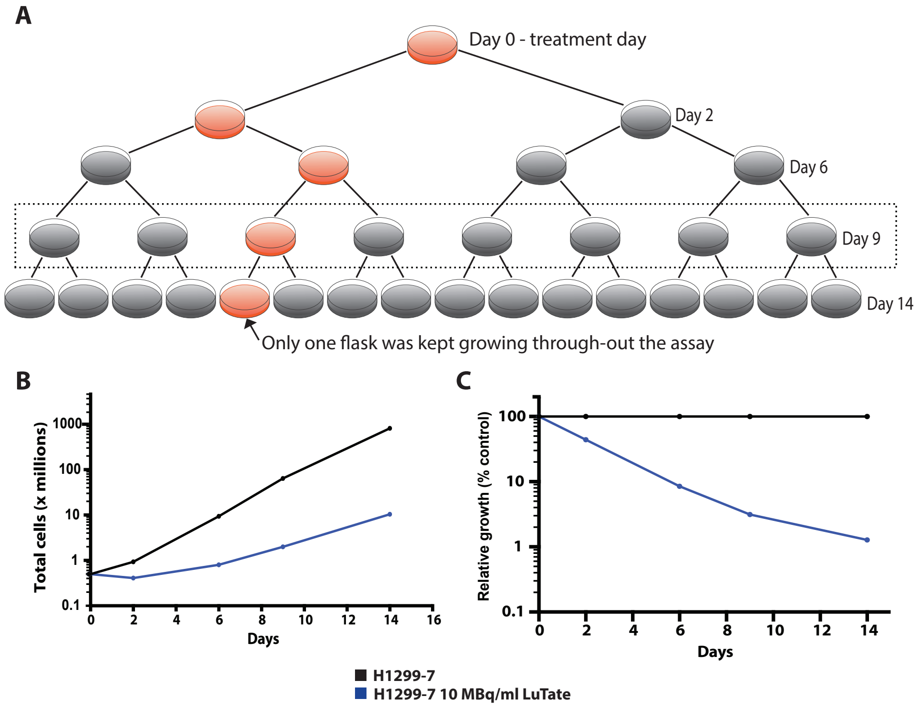

Supplementary Figure S2

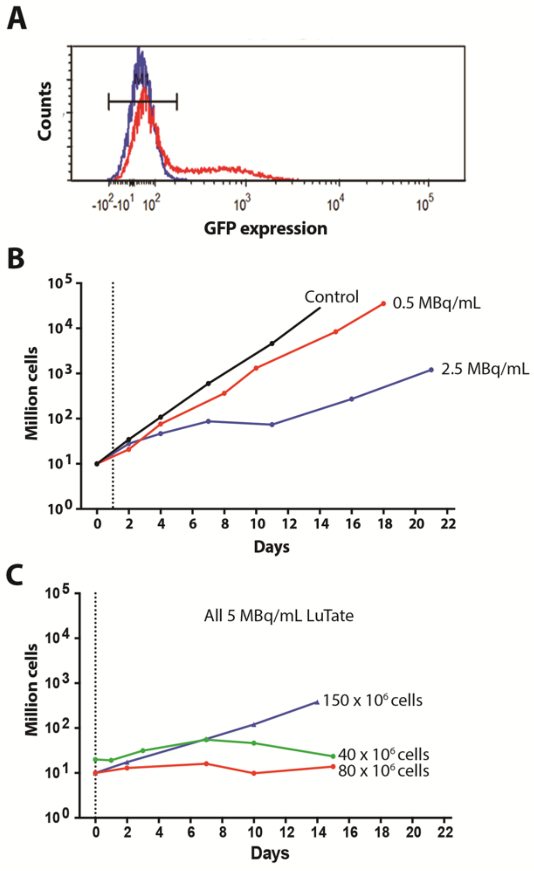

Supplementary Figure S3

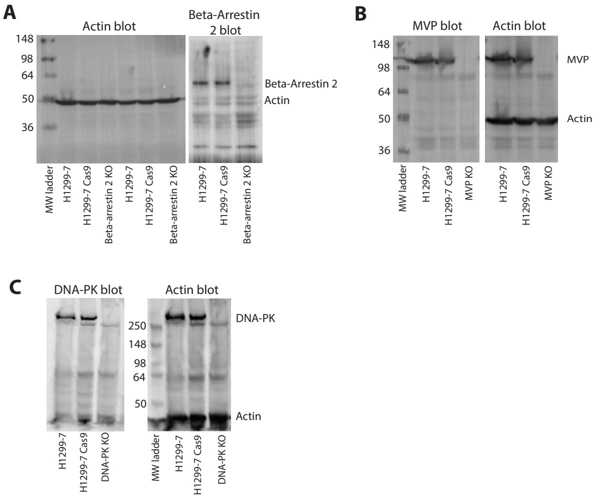

Supplementary Figure S4

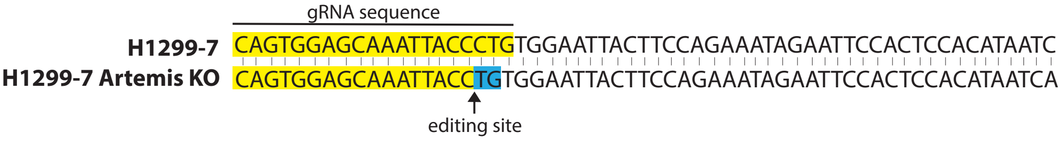

Supplementary Figure S5

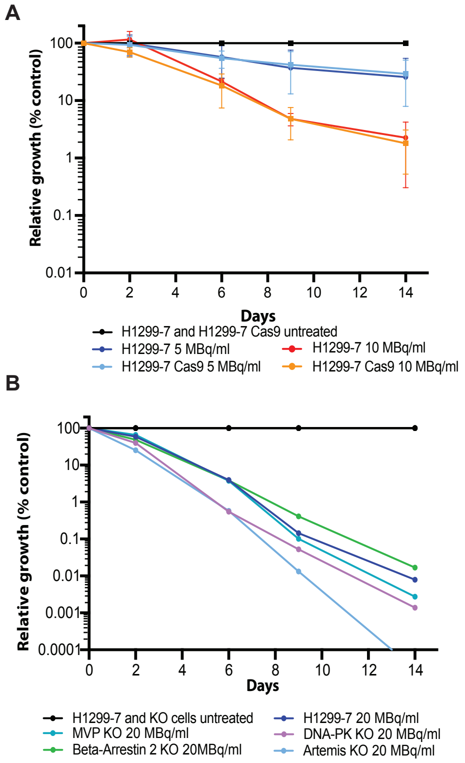

Supplementary Figure S6

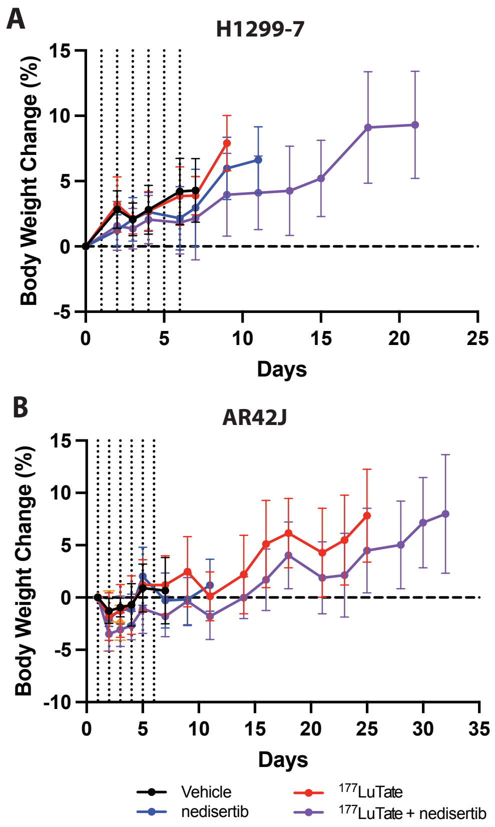

Supplementary Table 1

P5 Primer sequences

Name	Sequence
P5_0nt_stag_lentiGuide	AATGATACGGCGACCACCGAGATCTACACTCTTTCCCTACACGACGCTCTTCCGATCTTTGTGGAAAGGACGAAACAC*C*G
P5_1nt_stag_lentiGuide	AATGATACGGCGACCACCGAGATCTACACTCTTTCCCTACACGACGCTCTTCCGATCTCTTGTGGAAAGGACGAAACAC*C*G
P5_2nt_stag_lentiGuide	AATGATACGGCGACCACCGAGATCTACACTCTTTCCCTACACGACGCTCTTCCGATCTGCTTGTGGAAAGGACGAAACAC*C*G
P5_3nt_stag_lentiGuide	AATGATACGGCGACCACCGAGATCTACACTCTTTCCCTACACGACGCTCTTCCGATCTAGCTTGTGGAAAGGACGAAACAC*C*G
P5_4nt_stag_lentiGuide	AATGATACGGCGACCACCGAGATCTACACTCTTTCCCTACACGACGCTCTTCCGATCTCAACTTGTGGAAAGGACGAAACAC*C*G
P5_6nt_stag_lentiGuide	AATGATACGGCGACCACCGAGATCTACACTCTTTCCCTACACGACGCTCTTCCGATCTTGCACCTTGTGGAAAGGACGAAACAC*C*G
P5_7nt_stag_lentiGuide	AATGATACGGCGACCACCGAGATCTACACTCTTTCCCTACACGACGCTCTTCCGATCTACGCAACTTGTGGAAAGGACGAAACAC*C*G
P5_8nt_stag_lentiGuide	AATGATACGGCGACCACCGAGATCTACACTCTTTCCCTACACGACGCTCTTCCGATCTGAAGACCCTTGTGGAAAGGACGAAACAC*C*G

P7 Primer sequences

Name	Sequence to include	5'-3' Index read (rev comp sequence)	Full sequence
P7_index01_lentiGuide	CGGTTCAA	TTGAACCG	CAAGCAGAAGACGGCATACGAGATCGGTTCAAGTGACTGGAGTTCAGACGTGTGCTCTTCCGATCTTCTACTATTCTTTCCCCTGCACT*G*T
P7_index02_lentiGuide	GCTGGATT	AATCCAGC	CAAGCAGAAGACGGCATACGAGATGCTGGATTGTGACTGGAGTTCAGACGTGTGCTCTTCCGATCTTCTACTATTCTTTCCCCTGCACT*G*T
P7_index03_lentiGuide	TAACTCGG	CCGAGTTA	CAAGCAGAAGACGGCATACGAGATTAACTCGGGTGACTGGAGTTCAGACGTGTGCTCTTCCGATCTTCTACTATTCTTTCCCCTGCACT*G*T
P7_index05_lentiGuide	ATACTCAA	TTGAGTAT	CAAGCAGAAGACGGCATACGAGATATACTCAAGTGACTGGAGTTCAGACGTGTGCTCTTCCGATCTTCTACTATTCTTTCCCCTGCACT*G*T
P7_index06_lentiGuide	GCTGAGAA	TTCTCAGC	CAAGCAGAAGACGGCATACGAGATGCTGAGAAGTGACTGGAGTTCAGACGTGTGCTCTTCCGATCTTCTACTATTCTTTCCCCTGCACT*G*T
P7_index07_lentiGuide	ATTGGAGG	CCTCCAAT	CAAGCAGAAGACGGCATACGAGATATTGGAGGGTGACTGGAGTTCAGACGTGTGCTCTTCCGATCTTCTACTATTCTTTCCCCTGCACT*G*T
P7_index08_lentiGuide	TAGTCTAA	TTAGACTA	CAAGCAGAAGACGGCATACGAGATTAGTCTAAGTGACTGGAGTTCAGACGTGTGCTCTTCCGATCTTCTACTATTCTTTCCCCTGCACT*G*T
P7_index09_lentiGuide	CGGTGACC	GGTCACCG	CAAGCAGAAGACGGCATACGAGATCGGTGACCGTGACTGGAGTTCAGACGTGTGCTCTTCCGATCTTCTACTATTCTTTCCCCTGCACT*G*T
P7_index10_lentiGuide	TACAGAGG	CCTCTGTA	CAAGCAGAAGACGGCATACGAGATTACAGAGGGTGACTGGAGTTCAGACGTGTGCTCTTCCGATCTTCTACTATTCTTTCCCCTGCACT*G*T
P7_index14_lentiGuide	ATACTCGG	CCGAGTAT	CAAGCAGAAGACGGCATACGAGATATACTCGGGTGACTGGAGTTCAGACGTGTGCTCTTCCGATCTTCTACTATTCTTTCCCCTGCACT*G*T
P7_index16_lentiGuide	GCACAGTT	AACTGTGC	CAAGCAGAAGACGGCATACGAGATGCACAGTTGTGACTGGAGTTCAGACGTGTGCTCTTCCGATCTTCTACTATTCTTTCCCCTGCACT*G*T
P7_index17_lentiGuide	CGTGGATT	AATCCACG	CAAGCAGAAGACGGCATACGAGATCGTGGATTGTGACTGGAGTTCAGACGTGTGCTCTTCCGATCTTCTACTATTCTTTCCCCTGCACT*G*T
P7_index18_lentiGuide	TAGTAGAA	TTCTACTA	CAAGCAGAAGACGGCATACGAGATTAGTAGAAGTGACTGGAGTTCAGACGTGTGCTCTTCCGATCTTCTACTATTCTTTCCCCTGCACT*G*T
P7_index19_lentiGuide	GCACGATT	AATCGTGC	CAAGCAGAAGACGGCATACGAGATGCACGATTGTGACTGGAGTTCAGACGTGTGCTCTTCCGATCTTCTACTATTCTTTCCCCTGCACT*G*T

Supplementary Table 2

sgRNA sequences for CRISPR validation

Target		Sequence	Sense/Antisense		
Artemis	1	CAGTGGAGCAAATTACCCTG	sense	Human	sgRNA
	2	GGAGACTTCAGATTGGCGCA	sense	Human	sgRNA
DNA-PK	1	CTCCATAATCCGGACCACAA	antisense	Human	sgRNA
	2	GCAACATCAGAATACTATGG	sense	Human	sgRNA
MVP	1	ATCCCTCTAGACGAGAACGA	sense	Human	sgRNA
	2	CGTCATCACCATCGAAACGG	sense	Human	sgRNA
Beta-Arrestin 2	1	CACAGGACACTTGTACTGGG	antisense	Human	sgRNA
	2	CCAAAAGCTGTACTACCATG	sense	Human	sgRNA

Supplementary Table 3

Primer sequences for CRISPR editing confirmation

Target	Sequence	Sense/Antisense
Artemis	TCGTGTCTGGAAACTTTTCTACT	Sense
	GGTTTGGGAGTAGCATCCCC	Antisense
